# Caudal *Fgfr1* disruption produces localised spinal mis-patterning and a terminal myelocystocele-like phenotype in mice

**DOI:** 10.1242/dev.202139

**Published:** 2023-10-10

**Authors:** Eirini Maniou, Faduma Farah, Abigail R. Marshall, Zoe Crane-Smith, Andrea Krstevski, Athanasia Stathopoulou, Nicholas D. E. Greene, Andrew J. Copp, Gabriel L. Galea

**Affiliations:** Developmental Biology and Cancer Department, UCL Great Ormond Street Institute of Child Health, London WC1N 1EH, UK

**Keywords:** Neural tube, FGFR1, Terminal myelocystocele, Patterning

## Abstract

Closed spinal dysraphisms are poorly understood malformations classified as neural tube (NT) defects. Several, including terminal myelocystocele, affect the distal spine. We have previously identified a NT closure-initiating point, Closure 5, in the distal spine of mice. Here, we document equivalent morphology of the caudal-most closing posterior neuropore (PNP) in mice and humans. Closure 5 forms in a region of active FGF signalling, and pharmacological FGF receptor blockade impairs its formation in cultured mouse embryos. Conditional genetic deletion of *Fgfr1* in caudal embryonic tissues with *Cdx2^Cre^* diminishes neuroepithelial proliferation, impairs Closure 5 formation and delays PNP closure. After closure, the distal NT of *Fgfr1*-disrupted embryos dilates to form a fluid-filled sac overlying ventrally flattened spinal cord. This phenotype resembles terminal myelocystocele. Histological analysis reveals regional and progressive loss of SHH- and FOXA2-positive ventral NT domains, resulting in OLIG2 labelling of the ventral-most NT. The OLIG2 domain is also subsequently lost, eventually producing a NT that is entirely positive for the dorsal marker PAX3. Thus, a terminal myelocystocele-like phenotype can arise after completion of NT closure with localised spinal mis-patterning caused by disruption of FGFR1 signalling.

## Teaser

**Summary:** Conditional deletion of *Fgfr1* prevents formation of the posterior neuropore caudal closure site known as Closure 5, which delays spinal neural tube closure, and phenocopies the closed spinal dysraphism ‘terminal myelocystocele’ in mice.

## INTRODUCTION

Neural tube defects (NTDs) are a heterogenous group of congenital malformations resulting from dysmorphogenesis of the neural tube (NT), the embryonic precursor of the central nervous system. The most common and severe NTDs are those caused by failure to close the NT, including open spina bifida (myelomeningocele). Successful closure of the NT during primary neurulation requires coordinated deformation of the neural plate into a continuous tube of neuroepithelial cells covered by surface ectoderm along the back of the embryo (Nikolopoulou et al., 2017). Closure begins at distinct closure-initiating points, where the opposite neural folds are first brought into apposition at the dorsal midline. Five closure-initiating points had originally been proposed to initiate NT closure, based on anatomical clustering patterns of NTD lesions in human embryos (Van Allen et al., 1993; Martinez-Frias et al., 1996). Three closure-initiating points were subsequently experimentally identified in mouse embryos: Closure 1 at the hindbrain/cervical boundary; Closure 2 at the forebrain/midbrain boundary (absent in humans); and Closure 3 at the ventral forebrain (Copp and Greene, 2010). The regions of open NT between these closure points, called neuropores, close through progressive zippering (Nikolopoulou et al., 2017). No closure-initiating point is present where Closure 4 was initially inferred, but we have more recently confirmed the presence of Closure 5 at the end of the presumptive spinal NT in mice (Galea et al., 2017, 2018). Closure 5 is a load-bearing tissue structure that physically holds the neural folds together at the dorsal midline during an ∼10 h period from when it is first morphologically identifiable (∼25 somite stage) until the spinal NT is fully closed (∼30 somite stage). This ephemeral structure has not yet been visualised in human embryos.

In mice, surface ectoderm cells at Closure 5 extend cellular ruffles, consistent with zippering in a caudal-to-cranial direction (Galea et al., 2017). Thus, the presumptive spinal neuropore, called the posterior neuropore (PNP), closes primarily due to caudally directed zippering from Closure 1, biomechanically aided at late stages by Closure 5. After completion of PNP closure, the NT lumen extends caudally beyond Closure 5 through a process of mesenchyme-to-epithelium transition, with lumen formation referred to as secondary neurulation, forming the sacral and coccygeal spine. Several important but poorly understood NTDs characteristically localise to the junctional region between primary and secondary neurulation, where Closure 5 forms (Yang et al., 2022; Copp and Greene, 2013). These include terminal myelocystocele, often described as a ‘closed’ form of spina bifida because the dysmorphic spinal cord is covered with skin (Lee et al., 2020). Terminal myelocystocele characteristically presents as a trumpet-like flaring of the distal central canal of the spinal cord, forming a localised cystic dilation at the terminal end of the body that continues to expand postnatally (Lee et al., 2020; Pang et al., 2012). It is commonly associated with other malformations, including of musculoskeletal and urogenital structures, and variable neurological dysfunction (Pang et al., 2012).

There are, to our knowledge, no established animal models of terminal myelocystocele that mechanistically explain its pathogenesis. The most widely cited hypothesis for its formation relates to a balloon-like dilation observed at the junction between the primary and secondary NT of chick embryos (Lee et al., 2020), although this is an aspect of normal neurulation and is not a pathological finding. The joined primary and secondary NT form a continuous lumen bordered by neuroepithelial cells that differentiate into dorsoventrally patterned neural lineages. Many progenitor domains are similar between the primary and secondary NT, including a dorsal PAX3-expressing domain, intermediate PAX6 domain and ventral FOXA2 domain (Shum et al., 2010). However, there are regional differences in neural patterning, including the absence of motor neuron differentiation in the caudal NT region formed through secondary neurulation (Shum et al., 2010). Motor neuron progenitors (pMNs) differentiate in the ventral primary NT and are characteristically identified by expression of the transcription factor OLIG2, which is promoted by high levels of sonic hedgehog (SHH) morphogen from the NT floorplate and underlying notochord (Agius et al., 2004; Nishi et al., 2015). SHH is the best-established inducer of ventral NT fates (Dessaud et al., 2007; Balaskas et al., 2012), but does not act in isolation. For example, fibroblast growth factor (FGF) ligands promote maintenance of spinal neural progenitor identities while restricting differentiation into post-mitotic neurons (Diez del Corral et al., 2002; Lobjois et al., 2004; Diez et al., 2017). FGF signalling opposes somitic mesoderm-derived retinoic acid, which promotes neuron terminal differentiation in part by downregulating *Fgf8* (Ribes et al., 2009; Novitch et al., 2003; Diez del Corral et al., 2003). Loss of the retinoic acid-synthesising enzyme *Raldh2* does not prevent neuroepithelial commitment, as shown by SOX2 expression, but impairs differentiation of dorsoventral progenitor domains, including ventral OLIG2 and intermediate PAX6 in mice (Molotkova et al., 2005).

Insults that change the timing of neural progenitor differentiation have also been associated with open spina bifida in mice, such as loss of the dorsal neural tube marker PAX3 (Sudiwala et al., 2019; Epstein et al., 1991) or unrepressed SHH signalling causing expansion of ventral neural tube fates (Patterson et al., 2009; Qin et al., 2011). Studies into the interplay between neurulation and subsequent neurogenesis have been limited by the severely abnormal morphology of embryos lacking key mediators. For example, embryos globally lacking the FGF receptor FGFR1 die soon after gastrulation without closing any part of their NT (Yamaguchi et al., 1994). FGF pathway components, including ligands such as neuroepithelial FGF8 are particularly enriched in the caudal-most part of embryo, where Closure 5 forms (Gofflot et al., 1997).

Here, we used a combination of pharmacological antagonism and regional genetic deletion to test the contributions of FGF signalling, specifically through FGFR1, in PNP closure and Closure 5 formation. Conditional genetic deletion of *Fgfr1* produces mouse fetuses that are viable until birth but have a cystic dilation of the distal spinal NT that forms a closed spinal dysraphism. We find that the embryological origins of this malformation involve regional and progressive loss of NT ventral progenitor domains before the closed and dorsalised NT flattening ventrally, while retaining its dorsal skin covering, closely resembling terminal myelocystocele in humans.

## RESULTS

### Gradual neural fold elevation forms Closure 5 at late stages of spinal closure

**Fig. 1.**
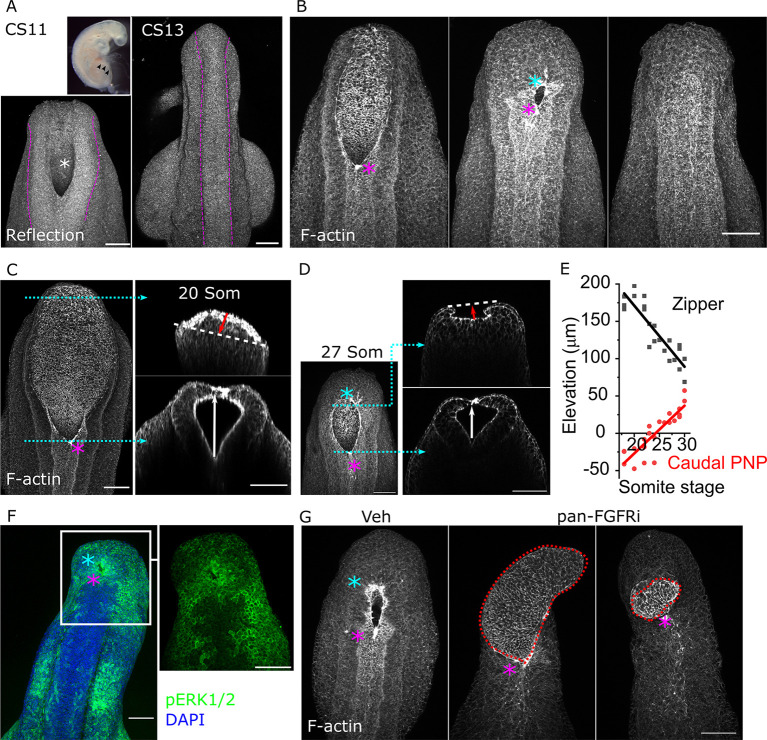
**FGF-dependent elevation of the distal neural folds forms Closure 5.** (A) Reflection confocal images of the PNP (asterisk and black arrowheads) of a CS11 human embryo and closed neural tube of a CS13 human embryo. Dashed magenta lines indicate the borders of the neuroepithelium. Scale bars: 50 µm. (B) Representative confocal images of phalloidin-stained mouse PNPs at sequential stages of closure (E9.5-E10.5), illustrating the formation of Closure 5 and completion of closure. Scale bar: 100 µm. (C) Confocal image of a phalloidin-stained PNP from a 20-somite stage mouse embryo showing optical cross-sections at the level of the zippering point and at 90% of the length from the proximal end of the PNP. White arrow indicates zippering point elevation; red arrow indicates eversion of the caudal neural folds relative to the apical surface of the neuroepithelium. (D) Equivalent to C showing a 27-somite stage mouse embryo PNP indicating elevation of the caudal neural folds. (E) Quantification of elevation at the zippering point and caudal neural folds (90% of the length of the PNP) in mouse embryos at the indicated somite stages. (F) Whole-mount confocal image of a 29 somite-stage mouse PNP stained for pERK1/2 immunolocalization. The two images were acquired using different objectives. Scale bars: 100 µm. (G) Confocal images of a vehicle-treated and two pan-FGFR inhibitor-treated mouse embryos after 24 h of whole-embryo culture. Dashed red lines indicate the abnormal neuroepithelium that protrudes out of the PNP in inhibitor-treated embryos. Scale bar: 100 µm. Magenta asterisks indicate the rostral zippering point; cyan asterisks indicate Closure 5.

Human primary neurulation is completed by Carnegie stage (CS) 13, around 30-35 days of gestation (Yang et al., 2014). At CS11, the PNP is open and has a spade-like morphology (Fig. 1A) equivalent to the mouse PNP on embryonic day (E) 9.5 (Fig. 1B). Progression of mouse PNP closure involves caudal narrowing to form an elliptical opening surrounded by purse string-like F-actin cables as Closure 5 forms at the caudal extreme of the PNP (Fig. 1B). Although late-stage human PNPs are not available to us for 3D imaging, bright-field images archived by the Human Developmental Biology Resource suggest equivalent changes in shape to form an elliptical PNP with caudal Closure 5 in humans as in mice (Fig. S1).

We have previously demonstrated that Closure 5 is a load-bearing structure (Galea et al., 2017, 2018). Here, we observe that it forms through gradual elevation of the caudal neural fold tips. The caudal PNP neuroepithelium is everted at earlier developmental stages, but progressively becomes apically concave as the neural fold tips elevate dorsally in embryos with more than 25 somites (Fig. 1C-E), which is when Closure 5 typically forms. Dorsal elevation of the rostral zippering point follows an inverse pattern: it is initially elevated ∼200 µm above the ventral-most apical neuroepithelium, but its elevation halves to ∼100 µm in later-stage embryos with established Closure 5 (Fig. 1E). The continuum of neural fold morphologies, from early eversion to late elevation, suggests coordinated morphogenesis of the caudal PNP to complete primary neurulation.

### Pharmacological blockade of FGF signalling diminishes Closure 5 formation

The caudal PNP is known to be enriched in components of cascades, including canonical Wnt and FGF signalling (Fig. S2). FGF components are particularly prominent (Fig. S2) and we observe marked phosphorylation of the putative downstream effector of the of the pathway, ERK1/2, selectively around Closure 5 (Fig. 1F). Phosphorylated FGFR1 is present diffusely around the PNP, particularly in the cytoplasm of mitotic cells (Fig. S3A), and its staining intensity is reduced in embryos treated for 24 h with the pan-FGFR inhibitor BGJ 398 (Fig. S3B). Pharmacological inhibition of FGFR signalling in mouse whole-embryo culture causes PNP morphology to become highly irregular, particularly in the caudal-most PNP where abnormal tissue outgrowth forms instead of Closure 5 (Fig. 1G). This inhibitor does not change somite gain (vehicle 27±3 somites, pan-FGFR inhibitor 26±3 somites at the end of culture, mean±s.d.), but produces larger somites (Fig. S4).

The range of PNP morphologies observed in FGFR-inhibited embryos suggests abnormal progression of closure (Fig. S5A). Cells within this outgrowth are derived from neuromesodermal progenitors that are lineage traced with *T^CreERT2^*, which continues to lineage trace both neural tube and mesoderm cells after 24 h of FGFR inhibition (Fig. S5B). Cells in the PNP outgrowth also continue to express the neuroepithelial marker N-cadherin (Fig. S5C).

**Fig. 2.**
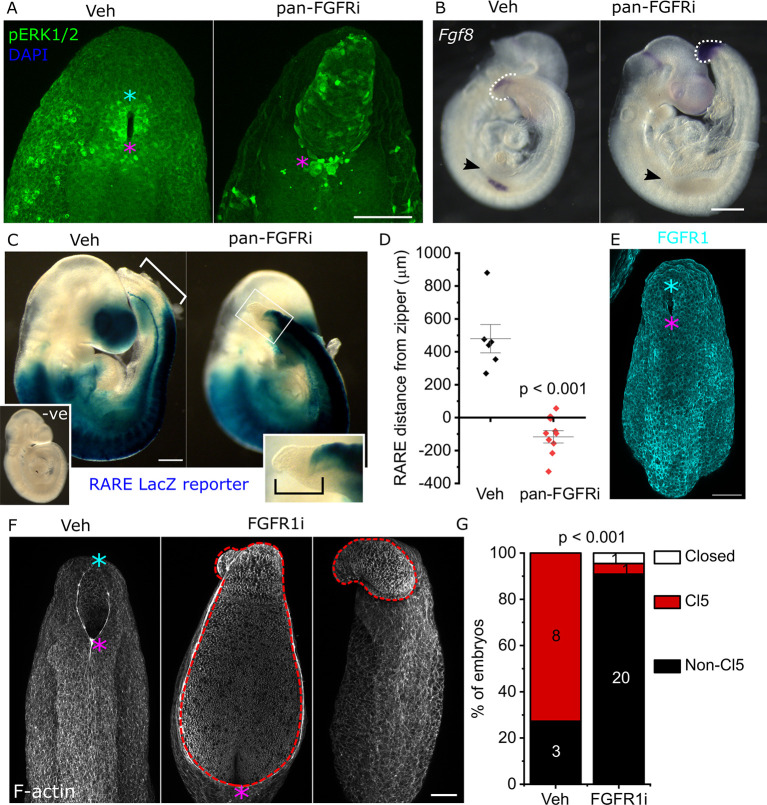
**FGF signalling is required for timely PNP closure.** (A) Whole-mount stained confocal images showing pERK1/2 immunolocalization in vehicle- and pan-FGFRi-treated embryos after 24 h of whole-embryo culture. pERK-bright cells persist around the PNP rim in the pan-FGFRi-treated embryo. Scale bar: 100 µm. (B) Whole-mount *Fgf8 in situ* hybridisation in vehicle- and pan-FGFRi-treated embryos after 8 h of whole-embryo culture. Dashed white lines indicate the end of the tailbud; arrows indicate the forelimb buds. Scale bar: 500 µm. (C) Bright-field images showing RARE-mediated LacZ expression domain in vehicle and pan-FGFRi-treated embryos after 24 h of whole-embryo culture. An equivalently processed negative (−ve) control embryo lacking LacZ is also shown. The white bracket indicates the distance between the RARE domain and the zippering point. The black bracket indicates that the RARE domain does not extend to the end of the PNP in pan-FGFRi-treated embryos. Scale bar: 350 µm. (D) Quantification of RARE domain distance to the end of zippering point in vehicle and pan-FGFRi-treated embryos after 24 h of whole-embryo culture. Individual points represent data from individual embryos; *P*-value was calculated using an unpaired *t*-test. (E) Whole-mount immunolocalization of Fgfr1 in a 28-somite stage embryo. Scale bar: 100 µm. (F) Confocal images of embryos treated with vehicle or a second Fgfr1-targeting antagonist after 24 h of whole-embryo culture. Dashed red lines outline the abnormal neuroepithelium in inhibitor-treated embryos. Scale bar: 100 µm. (G) Quantification of the proportion of embryos with closed PNPs, open PNPs with Closure 5 morphology (caudally narrowed PNP with elevated caudal neural folds that meet dorsally) or open PNPs without Closure 5 in vehicle and FGFR1-targeting inhibitor-treated embryos after 24 h of whole-embryo culture. Numbers indicate the number of embryos observed in each category. Magenta asterisks indicate the rostral zippering point; cyan asterisks indicate Closure 5.

Phosphorylation of ERK1/2 around the PNP rim persists despite FGFR inhibition (Fig. 2A), suggesting activation through other mechanisms, different phosphorylation stabilities or distinct regulatory feedback mechanisms. Expression of the pathway ligand *Fgf8* is retained or increased in the PNP neuroepithelium, whereas it is abolished in the forelimb bud of FGFR-inhibited embryos (Fig. 2B, Fig. S6A), indicating tissue-specific regulation. Retinoic acid signalling is known to interact mutually antagonistically with caudally active FGF signalling (Diez del Corral et al., 2003; Goldbeter et al., 2007). Pharmacological FGFR blockage causes caudal expansion of the retinoic acid signalling domain genetically labelled with a retinoic acid response element (RARE) that drives LacZ expression. RARE caudal expansion is observed within 8 h of FGFR inhibition, before the PNP is morphologically abnormal (Fig. S6B,C), and persists after 24 h of inhibition (Fig. 2C,D). However, RARE activity does not detectably extend to the caudal-most PNP region where Closure 5 should form (Fig. 2C). The caudal PNP fails to elevate, remaining everted, in contrast to vehicle-treated controls, which elevate their caudal PNP beyond the 25-somite stage (Fig. S7A,B). FGFR inhibitor-treated embryos largely fail to form Closure 5 and do not complete PNP closure by E10.5 (Fig. S7C).

Various FGF receptors are known to be expressed in and around the PNP, and may have both redundant and obligate roles. *Fgfr3* knockout causes postnatal phenotypes without NTDs (Colvin et al., 1996). *Fgfr2* embryos die at ∼E11.5, after successful closure of the neural tube (Xu et al., 1998). *Fgfr1* is expressed diffusely around the PNP rim (Fig. 2E) (Wahl et al., 2007) and its chimeric deletion causes distal spina bifida (Deng et al., 1997). We therefore focused on *Fgfr1* as the more likely non-redundant mediator of FGF signalling that promotes PNP closure. Treating cultured embryos with a second FGFR1-targeting pharmacological antagonist, PD 173074 (Mohammadi et al., 1998; Szymczyk et al., 2022), similarly caused neuroepithelial outgrowth, failure of caudal elevation and failure of Closure 5 formation (Fig. 2F,G). This second antagonist also did not change somite gain (at the end of culture: vehicle, 28±3 somites; FGFR1-targeting inhibitor, 29±2 somites; mean±s.d.) and unlike the previously used inhibitor it did not change somite size (Fig. S4).

### *Fgfr1* conditional deletion prevents Closure 5 formation, but does not stop spinal closure

**Fig. 3.**
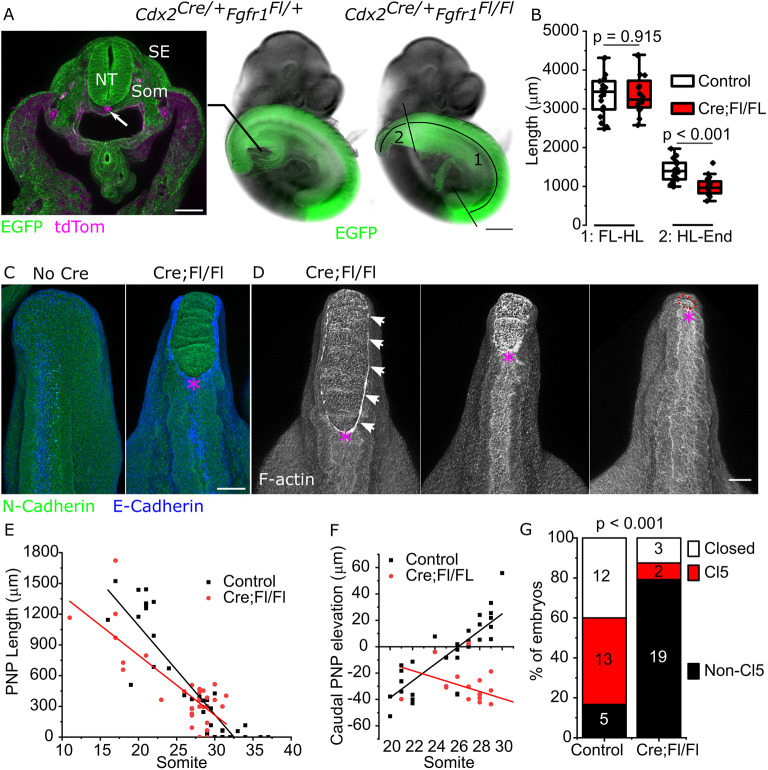
**Caudal genetic deletion of *Fgfr1* impairs Closure 5 formation and the progression of PNP closure without diminishing embryo viability.** (A) E10.5 embryos showing the *Cdx2^Cre^*-recombination domain lineage traced using mTmG (EGFP) as a confocal-imaged cross-section and bright-field images. NT, neural tube; Som somite; SE, surface ectoderm. 1 and 2 indicate the forelimb (FL) to hindlimb (HL) and hindlimb to tail end distances, respectively, quantified in B. Scale bars: 100 µm (confocal); 400 µm (bright field). (B) Quantifications of FL-HL and HL-tail end lengths in 28-31 somite stage embryos. (C) Confocal images of whole-mount immunofluorescently stained embryos showing E- and N-cadherin localization in E10.5 embryos with no Cre and Cre;Fl/Fl littermate. Scale bar: 100 µm. (D) Whole-mount images of three phalloidin-stained Cre;Fl/Fl embryos collected at E10.5, suggesting continued PNP closure. Scale bar: 100 µm. (E) Quantification of PNP length versus somite stage in embryos collected at E9.5-E10.5. (F) Quantification of caudal neural fold elevation (90% of PNP length) in embryos collected at E9.5-E10.5. (G) Quantification of the proportion of embryos collected at E10.5 with closed PNPs, open PNPs with Closure 5 morphology or open PNPs without Closure 5. Numbers indicate the number of embryos observed in each category. Magenta asterisks indicate the rostral zippering point. *P*-value calculated using Fisher's exact test.

Given inherent limitations of pharmacological antagonists, we sought to genetically delete *Fgfr1* in all tissues potentially involved in Closure 5 formation while avoiding early lethality seen in global knockouts (Brewer et al., 2015; Xu et al., 2002). This was achieved using paternally inherited CDX2P-NLS Cre (Hinoi et al., 2007) (henceforth *Cdx2^Cre^*) driving deletion of a previously reported (Xu et al., 2002) *Fgfr1* conditional allele (*Fgfr1^Fl/Fl^*). *Cdx2^Cre^* recombines in all embryonic tissues caudal to the cervical spine with some notable exceptions: at E10.5 it does not recombine in the notochord or lateral plate mesoderm (Fig. 3A,B). Heterozygous *Cdx2^Cre/+^Fgfr1^Fl/+^* (henceforth, Cre;Fl/+) mice are viable and fertile. Embryos with this genotype are morphologically indistinguishable from their Cre-negative littermates and are therefore included as ‘controls’. Homozygous *Cdx2^Cre/+^Fgfr1^Fl/Fl^* (henceforth, Cre;Fl/Fl) embryos appear caudally truncated, with an open PNP at E10.5 (Fig. 3A,C). Loss of *Fgfr1* does not impair embryo growth between the forelimb and hindlimb buds, but selectively truncates caudal elongation from the hind limbs to the end of the body (Fig. 3B). At this stage their PNP is irregularly shaped with corrugated neuroepithelium (Fig. 3C,D), reminiscent of FGFR antagonist-treated embryos in culture.

*Fgfr1* conditional deletion significantly diminishes proliferation of neuroepithelial cells, but not of mesodermal or hindgut cells, and does not substantially alter apoptosis in these tissues in the distal trunk of E10.5 embryos (Fig. S8A-E). To further explore the impact of *Fgfr1* loss of neuroepithelial proliferation, we analysed embryos at early (E9.5) and late (E10.5) stages of PNP closure. *Fgfr1* conditional deletion diminished the number of mitotic figures in the neuroepithelium at both timepoints (Fig. S8F,G). Cre;Fl/Fl embryos continue to assemble long F-actin cables bordering the PNP, as seen in wild-type embryos, and achieve progressive shortening of the PNP (Fig. 3D,E). The caudal PNP elevates progressively in control embryos, whereas in Cre;Fl/Fl it tends to become increasingly everted (Fig. 3F). Consequently, in embryos with more than 25 somites, 43% of control embryos have a PNP morphology indicative of Closure 5, compared with only 8.3% of Cre;Fl/Fl embryos (Fig. 3G). Thus, caudal deletion of *Fgfr1* causes morphologically abnormal PNPs that typically lack Closure 5 formation. At this stage of development, the domain of *Fgf8* expression appears expanded in Cre;Fl/Fl embryos compared with littermate controls (Fig. S9A), as had been seen after pharmacological FGFR inhibition.

### *Fgfr1* disruption causes a terminal myelocystocele-like phenotype

**Fig. 4.**
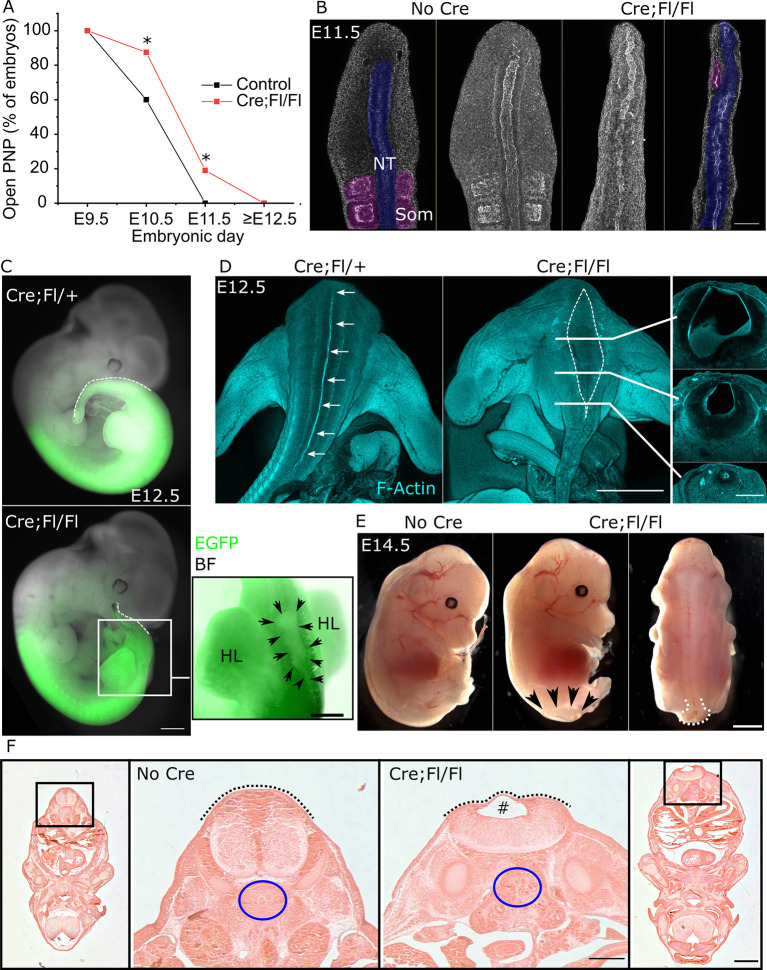
**Regional deletion of Fgfr1 causes delayed PNP closure and localized terminal dilation of the neural tube central canal.** (A) Proportion of control and Cre;Fl/Fl embryos collected with open spinal neural tubes at the days indicated. *P<0.05 by Fisher's exact test. (B) Whole-mount maximum projections flanked by optical cross-sections through the caudal end of a control and Cre;Fl/Fl littermate showing the neural tube (NT) and adjacent somites (Som). Scale bar: 100 µm. (C) Bright-field images showing the *Cdx2^Cre^* recombination domain (green, EGFP) at E12.5 in a Cre;Fl/+ control and Cre;Fl/Fl littermate. The dashed white line indicates the dorsal tail border. Black arrows in the enlargement outline the cystic neural tube lumen. Scale bars: 300 µm (whole embryo); 150 µm (enlargement). BF, bright field; HL, hindlimb. (D) Whole-mount phalloidin-stained embryos showing the continuous, narrow neural tube lumen (arrows) in the control and dilated lumen (dashed white line) in the Cre;Fl/Fl embryo. Solid lines indicate the positions of sections taken through the same Cre;Fl/Fl embryo. Scale bars: 1 mm (whole mount) and 250 µm (sections). (E) Bright-field images of a control and littermate Cre;Fl/Fl fetuses collected at E14.5. Black arrows indicate the extent of the spinal lesion in the Cre;Fl/Fl embryo; white dashed line indicates its sac-like dilation. Scale bar: 750 µm. (F) Haematoxylin and Eosin-stained sections through control and Cre;Fl/Fl littermates collected at E14.5. Dashed black lines indicate continuous skin covering, # indicates cystic dilation of the spinal cord lumen; blue circles indicate the ventral vertebral body in the control, which is absent in the Cre;F/Fl. Scale bars: 300 µm (low magnification); 100 µm (high magnification).

A significantly smaller proportion of Cre:Fl/Fl than control embryos achieves PNP closure by E10.5 and a subset remains open at E11.5 (Fig. 4A), exhibiting open lesions caudal to the hind limb buds (Fig. S9B), whereas all control embryos close their PNP by this timepoint (Fig. 4B). All fetuses collected from E12.5 onwards had closed neural tubes (Fig. 4A), defined as surface ectoderm or skin fully covering the developing spinal cord. Control embryos at E11.5 have a closed quasi-cylindrical neural tube extending past paired somites into the tail bud (Fig. 4B). Somites flanking the closed neural tube within the embryonic tail are markedly hypoplastic in Cre;Fl/Fl embryos (Fig. 4B). Hypoplastic somites may result in impaired synthesis of retinoic acid lateral to the neural tube as these structures normally express the retinoic acid-synthesising enzyme RALDH2 (Fig. S10A).

At E12.5, Cre:Fl/Fl embryos develop a cystic dilation of the neural tube lumen selectively between the hindlimbs (Fig. 4C,D). Serial cross-sections reveal a trumpet-like flaring of the central canal with a thin dorsal neuroepithelium overlying the dilated region (Fig. 4D). By E14.5, the dorsal thinning of the roof plate and canal dilation progresses to form a ventrally flattened spinal cord that has an ‘open’ morphology, yet is covered by skin overlying a fluid-filled sac (Fig. 4E,F). This sac-like lesion at the end of the spinal cord morphologically resembles terminal myelocystocele lesions. Additional malformations are evident in these fetuses, including absence of the ventral vertebral body (Fig. 4F), limb malformations and an apparent delay in perineal fusion (Fig. S10B,C).

**Fig. 5.**
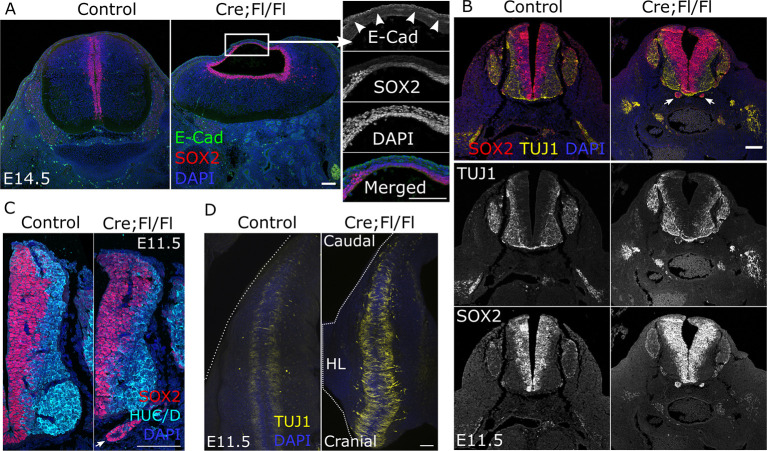
**Neuroepithelial commitment to post-mitotic neurons is not diminished by loss of *Fgfr1*.** (A) Immunofluorescent localization of the neuroepithelial marker SOX2 and epidermal marker E-cadherin (arrowheads) through the distal spinal cord of a control and Cre;Fl/Fl fetus. Scale bars: 100 µm. (B,C) Progenitor (SOX2) and committed neuron (B, TUJ1; C, HUC/D) immunofluorescence of sections through the low-lumbar spinal cord of a control and Cre;Fl/Fl embryo collected at E11.5. There are ectopic clusters of SOX2-positive cells below the neural tube of the Cre;Fl/Fl embryo (white arrows, described below). Scale bar: 100 µm. (D) Whole-mount confocal images showing TUJ1 immunolocalization in the dorsal aspect of a control and littermate Cre;Fl/Fl embryo collected at E11.5 and imaged equivalently. Dashed white lines indicate the body outline. HL, hindlimb. Scale bar: 100 µm.

We immunolabelled neural progenitors to confirm the identity of cells in the mis-shapen spinal cord of Cre;Fl/Fl embryos. SOX2 labels a continuous neuroepithelial layer encircling the central canal, but this layer is thinned dorsally in Cre;Fl/Fl embryos at E14.5 (Fig. 5A). This dorsal neuroepithelium lies ventral to the overlying skin layer, which stains positively for E-cadherin (Fig. 5A). This dysmorphology arises in a region of closed neural tube between the hindlimb buds that is morphologically normal at E11.5 (Fig. 5B). At this stage, HUC/D and TUJ1-positive neurons are specified in both control and Cre;Fl/Fl embryos (Fig. 5B-D). The ratio of SOX2-positive to HUC/D-positive cells along the lateral neural tube is not significantly different between control and Cre;Fl/Fl embryos (Fig. S11A,B). Dorsal views of the neural tube show distinct parallel populations of TUJ1-positive neurons extending projections between the hind limb buds in both, but more prominently in Cre;Fl/Fl than control embryos (Fig. 5D) potentially due to hypoplasia of surrounding tissues making these neurons more readily visible.

### Regional neural tube dorsalisation precedes cystic dilation

FGF signalling has previously been found to maintain neuroepithelial cells in a progenitor state while reducing their terminal differentiation (Diez et al., 2017). Cross-sections through the lumbar neural tube of Cre;Fl/Fl embryos at E11.5 reveal distinct ventral (SHH, FOXA2 and OLIG2; Fig. S12A,B) and dorsal (PAX3; Fig. S12C) progenitor domains within the neural tube. Migrating neural crest cells, labelled with SOX9, can readily be identified in streams lateral to the neural tube of Cre;Fl/Fl embryos (Fig. S12C). PAX3 also immunolabels paraxial mesoderm (dermomyotome), which is markedly smaller in *Fgfr1*-disrupted embryos (Fig. S12C).

**Fig. 6.**
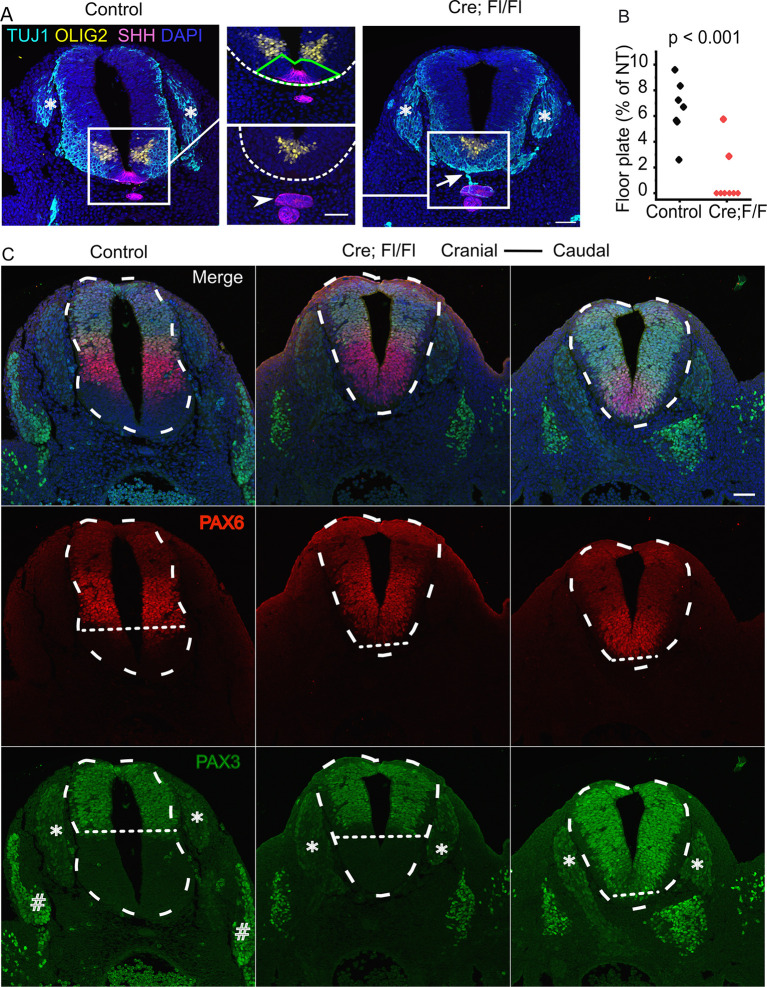
**Localized progressive loss of ventral spinal progenitor domains precedes cystic dilation of the neural tube central canal in Fgfr1-disrupted embryos.** (A) Immunofluorescent localization of the pMN marker OLIG2, floorplate marker SHH and neuron marker TUJ1 through the lumbar spinal cord of a control and Cre;Fl/Fl littermate collected at E11. White boxes indicate the regions shown at higher magnification; dashed white lines border the ventral neural tube; green polygon indicates the region ventral to the OLIG2 domain quantified in B; asterisks indicate the dorsal root ganglia; arrowhead indicates ectopic Shh-expressing tissue; arrow indicates TUJ1-positive projection from the ectopic tissue. Scale bars: 50 µm (higher magnification). (B) Quantification of the region of the neural tube ventral to the OLIG2 domain (‘floor plate’) as a proportion of neural tube area in control and Cre;Fl/Fl embryos collected at E10.5 and sectioned between the hindlimb buds. (C) Immunofluorescent localization of the intermediate neural progenitor marker PAX6 and dorsal/neural crest marker PAX3 in E11.5 embryos. PAX3 also labels cells in the neural crest-derived dorsal root ganglia (asterisk) and somite-derived dermomyotome (#). Dashed white lines outline the neural tube; dotted lines indicate the ventral extent of each domain. Serial sections in the Cre;Fl/Fl embryos are ∼50 µm apart. Scale bar: 50 µm.

*Fgfr1* disruption causes progressive abnormalities of spinal progenitor domains. Cre;Fl/Fl embryos commonly have ectopic clusters of cells ventral to and distinct from the neural tube. These clusters express SOX2 (Fig. 5B,C), confirming their neuroepithelial identity, as well as SHH (Fig. 6A) and FOXA2 (Fig. S12B), and appear to produce TUJ1-positive axonal projections (Fig. 6A). They are identifiable in a short region of the neural tube between the hindlimbs and may act as an ectopic source of SHH when present alongside the floorplate and notochord (Fig. S12D). However, SHH-expressing ectopic clusters are also observed adjacent to locations devoid of detectable SHH in the ventral neural tube (Fig. 6A).

Regional loss of floor-plate markers is observable in *Fgfr1*-disrupted embryos as early as E10.5 (Fig. 6B), initially replaced by ventral extension of the OLIG2 domain between the hindlimb buds (Fig. S12E). By E11.5, even the OLIG2 domain is progressively lost as the intermediate PAX6 domain expands ventrally (Fig. 6C). At more caudal levels within the same embryo, the dorsal PAX3 domain extends along the entire dorso-ventral axis of the NT (Fig. 6C). PAX3 immunolabels the entire NT as it becomes abnormally circular in shape more caudally (Fig. S13A). Thus, ectopic ventral neuroepithelial clusters and progressive loss of ventral neural progenitor identities precedes NT dysmorphology, which produces a terminal myelocystocele-like cystic dilation of the lumen predictably at the terminal end of the embryo (Fig. S13B).

## DISCUSSION

The embryological origins of closed NTD subtypes have largely been inferred from their post-natal morphology and location. Animal models have been invaluable in identifying genetic/teratogenic causes of open NTDs (van Straaten and Copp, 2001), testing putative preventative agents (Greene and Copp, 2005) and even paving the way for spina bifida fetal surgery (Meuli et al., 1995), but such models are lacking for most cases of closed NTDs. Here, we show that the human closing PNP is morphologically analogous to that of mouse embryos, and provide bright-field images suggesting that it similarly adopts an elliptical structure with terminal Closure 5 at late stages of closure. The seemingly linear elevation of the caudal PNP neural folds with advancing somite stage suggests Closure 5 formation is a coordinated shape change starting at least 1 day earlier in mouse development.

We had previously reported impaired caudal neural fold elevation and abnormal Closure 5 associated with a caudal tissue ‘bulge’ in a model of conditional *Vangl2* deletion (Galea et al., 2018). We now observe a more exophytic outgrowth of caudal neuroepithelium where Closure 5 should have formed in embryos with pharmacologically-inhibited FGF signalling. Although we do not provide a definitive explanation for this outgrowth phenotype, it may involve overactivation of retinoic acid signalling, compensatory upregulation of pathway components, as indicated by increased *Fgf8* seen in our pharmacological and genetic models, and/or altered interactions between the neuroepithelium and paraxial mesoderm. After gastrulation, mesoderm and neuroepithelium share a *T*-expressing neuromesodermal progenitor population. FGF signalling has been suggested to maintain and direct differentiation of this progenitor pool (Goto et al., 2017; Turner et al., 2014). However, we do not observe gross cessation of contributions from this lineage to either neuroepithelial or mesodermal tissues within a 24 h period of FGF antagonism in whole-embryo culture.

Neuromesodermal progenitors contribute to axial elongation (Sambasivan and Steventon, 2020), for which FGF signalling is crucially required (Olivera-Martinez et al., 2012). This is consistent with our observation of caudal truncation in embryos with caudally deleted *Fgfr1*, suggesting a non-redundant role for this receptor in axial elongation beyond the hindlimbs. Diminished neuroepithelial proliferation observed after deletion of *Fgfr1* is consistent with the known mitogenic effects of this pathway on neural progenitors (Pearson et al., 2011; Murphy et al., 1990). Truncal elongation is unaffected despite being within the recombination domain of *Cdx2^Cre^*, suggesting receptor redundancy or activation of compensatory mechanisms that are well described for this complex pathway (Minowada et al., 1999). The concordance between failure of caudal PNP elevation and lack of Closure 5 formation seen with pharmacological antagonism of FGF receptors and selective genetic deletion of *Fgfr1* corroborates our hypothesis that completion of PNP closure selectively requires this receptor. The neural tube phenotypes we observe when using two different FGFR-targeting antagonists are very similar, but only the pan-FGFR inhibitor produces larger somites. An increase in somite size had previously been reported after pan-FGFR inhibition in chick embryos (Dubrulle et al., 2001). Absence of this somite phenotype when mouse embryos are cultured in the FGFR1-targeting antagonist used here may reflect differential tissue penetration, compound duration of action or FGFR1-independent mechanisms in mice. The latter explanation is consistent with our observations of smaller somites in embryos with genetic deletion of *Fgfr1* at later stages.

FGFR pharmacological antagonists are also known to have off-target effects, including those on VEGF receptors and SRC. However, both the antagonists used in this study and *Fgfr1* genetic deletion produced highly corrugated neuroepithelium at late stages of PNP closure. In-folding might produce the clusters of neuroepithelial cells observed in cross-section after the neural tube has closed in our *Fgfr1*-deletion model. Identifying the specific cell types in which *Fgfr1* signalling promotes neural fold elevation will require future tissue-specific deletion studies. Previous studies in which *Fgfr1* was deleted using *T^Cre^* reported musculoskeletal phenotypes comparable with those observed here, but not PNP dysmorphology or later terminal myelocystocele-like phenotypes (Wahl et al., 2007; Verheyden et al., 2005). Similarly, *Pax3^Cre^* deletion of *Fgfr1* caused kidney malformation, but neural tube defects were not reported (Poladia et al., 2006). It is not clear whether neural tube phenotypes were carefully assessed in those studies. *Cdx2^Cre^* used in this study provides near-global gene deletion in caudal embryonic tissues that starts sufficiently late in development to avoid lethality, yet sufficiently early to impact completion of neural tube closure. We observe that *Cdx2^Cre^* does not recombine in the notochord: whether persistent FGFR1 signalling in this tissue contributes to the phenotypes observed remains to be determined.

The ability of embryos with caudally deleted *Fgfr1* to achieve delayed yet complete PNP closure is surprising, not only because of the level of dysmorphology their closure process overcomes, but also because alternative disruptions of the same gene cause open spina bifida in mice. Chimeric embryos containing *Fgfr1*-null cells develop spina bifida in some cases (Deng et al., 1997). Embryos in which exon 3 of *Fgfr1* is globally deleted, preventing expression of the *Fgfr1α* isoform, also develop fully penetrant spina bifida, confounded by severe dysmorphology and early lethality (Xu et al., 1999). A hypomorphic *Fgfr1* allele has also been reported to cause spina bifida and skeletal malformations similar to those reported here (Pearson et al., 2011). In the current study, we deleted exons 8-14 (Xu et al., 2002), which include the transmembrane domain common to all isoforms (Gong, 2014), yet only produced closed spinal lesions at late stages of development. The open spinal neural tube we observed in a subset of embryos at E11.5 suggests predisposition to open spina bifida in our model that is not present in the environment and genetic background tested. A shared genetic basis between open and closed NTDs has previously been reported in the cranial region for exencephaly and encephalocele (Rolo et al., 2019).

Closed NTD-like phenotypes have been reported in a few other models. Deletion of the FGFR1 receptor-specific substrate (Frs) causes similar dilation of the distal neural tube in mouse embryos (Hoch and Soriano, 2006). A small localised dilation of the closed neural tube at the level of the hindlimb buds, but not terminal myelocystocele-like phenotypes at later stages, was previously observed in *Fgf3* knockout embryos (Anderson et al., 2016). At the opposite extreme, a more extensive dilation of the central canal of the spinal cord extending caudally from the thoracic spine has been described in noggin (*Nog*) knockout fetuses (Stottmann et al., 2006). Noggin is an antagonist of BMP signalling, which promotes dorsal spinal progenitor fates. Evidence of dorsoventral patterning disruption in *Nog^−/−^* embryos includes ventral expansion of PAX3 (McMahon et al., 1998). Another striking similarity between *Nog^−/−^* and *Fgfr1*-disrupted embryos in the current study is the preferential loss of dorsoventral patterning at the level of the hindlimb buds, although *Nog^−/−^* embryos also have markedly smaller neural tubes, which makes direct comparisons difficult (McMahon et al., 1998). One possible explanation for this regional phenotype is interactions with adjacent mesodermal structures, which are particularly abnormal caudally in our model. This is consistent with a lack of sclerotome-derived vertebral bodies below the cystic lesion of *Fgfr1*-disrupted fetuses. Sclerotome has been suggested to act both as a conduit for SHH diffusion as well as being a target tissue for its action (Kahane and Kalcheim, 2020), and somite-derived retinoic acid is mutually antagonistic with FGF signalling in regulating caudal neurogenesis (Diez del Corral et al., 2002). The combination of diminishing retinoic acid production by somites adjacent to the closed neural tube, and loss of repression by FGF signalling, suggests complex changes in the source, timing and intensity of neuroepithelial retinoic acid exposure in our model.

The transition from neurulation to subsequent neurogenesis continues to be explored. Premature neuronal differentiation and expansion of ventral spinal progenitor domains have been associated with open NTDs (Sudiwala et al., 2019; Patterson et al., 2009). One model of ventral progenitor domain expansion, deletion of the mitochondrial protein *Fkbp8*, produces extensive cystic dilation of the neural tube along the entire thoracic and lumbar spine (Wong et al., 2008). Histological sections of these *Fkbp8*-deficient fetuses with ventralised neural tubes suggests thinning of the ventral spinal cord, whereas the dorsalised neural tubes of our *Fgfr1*-disrupted embryos become thinned dorsally. The mechanisms for these changes in shape are unknown, but we propose that they cause characteristic spinal dysmorphology in conditions such as terminal myelocystocele.

The multiple interacting tissues and ubiquitous roles of FGF signalling present challenges in dissecting the molecular mechanisms by which terminal myelocystocele-like lesions emerge after closure of the neural tube. Here, we characterise a new model with which to begin addressing these questions. Dysmorphic PNP closure, ectopic neuroepithelial cluster formation that may act as aberrant Shh sources, diminished neuroepithelial proliferation during neurulation, progressive neural tube dorsalisation and hypoplasia of surrounding mesodermal structures, all precede trumpet-like flaring of the neural tube central canal in this model. We propose that, whereas failure of neurulation causes open NTDs, abnormalities in neural patterning contribute to the spectrum of clinically recognised closed NTDs.

## MATERIALS AND METHODS

### Animal procedures

Studies were performed under the regulation of the UK Animals (Scientific Procedures) Act 1986 and the National Centre for the 3Rs’ Responsibility in the Use of Animals for Medical Research (2019). C57BL/6 mice were bred in house and used as plug stock from 8 weeks of age. Mice were mated overnight, and the next morning was considered to be E0.5 if a plug was found. Alternatively, mice were mated for a few hours during the day, and the following midnight was considered to be E0.5. Pregnant females were sacrificed when their embryos were between E9.5 and E16.5.

*Fgfr1^Fl/+^* mice were as previously described (Xu et al., 2002). *Cdx2^Cre/+^* mice (Hinoi et al., 2007) were used to breed *Cdx2^Cre^Fgfr1^Fl/+^* stud males. The stud males were then crossed with *Fgfr1^Fl/Fl^* females to obtain *Cdx2^Cre^Fgfr1^Fl/Fl^* embryos. *Cdx2^Cre^Fgfr1*^Fl/+^ and Cre-negative embryos were phenotypically normal and considered littermate controls. *Rosa26-mTmG* reporter mice were as previously described (Muzumdar et al., 2007). For lineage tracing, *Cdx2^Cre^Fgfr1^Fl/+^* stud males were crossed with *Fgfr1^Fl/Fl^mTmG* females.

To lineage-trace neuromesodermal progenitors during FGFR pharmacological inhibition, *T^CreERT2/+^* stud males (Anderson et al., 2013) were crossed with *mTmG* females and tamoxifen (10 mg/mouse) was administered orally at 6 pm on E8.5 as previously validated (Savery et al., 2020). Embryos were then explanted into whole embryo culture starting at E9.5 and fixed 24 h after the start of culture. The RARE-hsp68LacZ reporter mice (JAX stock, 008477) (Rossant et al., 1991) were on a CD1 background as previously described (Rossant et al., 1991).

### Human embryos

One CS11 and one CS13 human embryo collected contemporaneously with this project were provided by the Human Developmental Biology Resource tissue bank (https://www.hdbr.org/; project registration number 200537. These embryos were non-destructively imaged using reflection confocal microscopy and returned to the tissue bank. Bright-field images of CD9-CS12 embryos collected by the tissue bank were also provided. These were identified from archival annotations stored by the tissue bank but were not available for further analysis.

### Embryo culture

Whole-mouse embryo culture was performed in neat rat serum following published protocols (Pryor et al., 2012). Pharmacological inhibitors pan-FGFRi (BGJ 398, Generon, used at a final concentration of 1 µM) or FGFR1-targeting inhibitor (PD 173074, Cambridge Bioscience, used at 1 µM) were dissolved in DMSO and added to the rat serum at the start of culture. For control conditions, 0.1% DMSO was added to the rat serum. Embryos were size matched and randomised to inhibitor or vehicle groups using a coin toss.

### Immunofluorescence

Embryos were dissected from their extra-embryonic membranes, rinsed in ice-cold PBS and fixed in 4% PFA overnight. Whole-embryo immunostaining was as previously described (Galea et al., 2017). Primary antibodies were used at 1:100 dilution and were as follows: rabbit E-cadherin (3195, Cell Signaling Technology), mouse N-cadherin (14215S, Cell Signaling Technology), rabbit pERK1/2 (9101, Cell Signalling Technology), mouse FGFR1 (ab824, Abcam) mouse anti-RALDH2 (sc-166362, Santa Cruz) and rabbit anti pFGFR1 (Tyr 653/654, GTX133526, Stratech).

For N-cadherin staining, antigen retrieval was first performed for 25 min at 100°C using 10 mM sodium citrate with 0.05% Tween 20 (pH 6.0). For pERK staining, a methanol/acetone post-fix step was added after PFA fixation. Embryos were washed with ice-cold 50:50 methanol/acetone for 30 min, followed by decreasing concentrations of methanol (10 min each) before blocking in 5% BSA solution in PBS with 0.1% Triton X-100. Secondary antibodies were used in 1:200 dilution and were Alexa Fluor conjugated (Thermo Fisher Scientific, A-11001, A48257, A32728, A-11057, A-11036, A-11034 and A78957). Alexa Fluor 568- and 647–conjugated phalloidin were from Thermo Fisher Scientific (A12380 and A22287).

For immunostaining of paraffin wax-embedded sections, antigen retrieval was always performed before blocking in 5% BSA solution. Primary antibodies were applied overnight at 4°C (1:100-1:200 dilution). Primary antibodies were as follows: rabbit anti-SOX2 (ab97959, Abcam), rabbit anti-SOX9 (7H13L8, Thermo Fisher Scientific), mouse anti-TUJ1 (MMS-435P, BioLegend), goat anti-OLIG2 (AF2418, Novus Biologicals), rabbit anti-SHH (C9C5, Cell Signalling Technology), mouse PAX3 (Pax3-c, DSHB), rabbit anti-PAX6 (901301, BioLegend), goat anti-FOXA2 (AF2400, R&D Systems) and mouse anti-HuC/D (16A11, Thermo Fisher). Sections were washed, incubated with the secondary antibodies listed above (1:500 dilution) and counterstained with DAPI for 2 h at room temperature.

### Confocal microscopy and image analysis

Images were captured on a Zeiss Examiner LSM 880 confocal using 10×/NA 0.5 or 20×/NA 1.0 Plan Apochromat water immersion objectives. Images were processed with Zen 2.3 software and visualised as maximum projections in Fiji (Schindelin et al., 2012). The inset in Fig. 1F was obtained with AiryFast scanning mode using the 20×/NA 1.0 water immersion objective. Reflection images of CS11 and CS13 human embryos were captured at 633 nm wavelength using a 10×/NA 0.5 water immersion objective. The *z* stacks were 3D rotated and visualised as maximum projections. Sections were captured with AiryFast scanning mode using the 10×/NA 0.5 dipping objective. Salt and pepper noise was removed when appropriate using the de-speckle or ‘remove outliers’ function in Fiji. ‘Surface subtraction’ was used to extract surface signal in whole-mount images using an in-house macro as previously described (Galea et al., 2018). This macro is available courtesy of Dr Dale Moulding on GitHub (https://github.com/DaleMoulding/Fiji-Macros).

For morphometric analysis, PNP length and width were calculated by annotating the PNP rim, and then measuring the major and minor axis using the fit ellipse function in Fiji. Neural fold elevation was measured in optical re-slices of confocal *z*-stacks. Distances between the limb buds and the caudal end of the embryos were measured in stereoscope images using the segmented line tool in Fiji. Floor-plate area was measured using the polygon tool and is expressed as percentage of total neural tube area.

### Histology and X-gal staining

After overnight fixation in 4% PFA, E11.5 embryos were dehydrated with increasing concentrations of ethanol (20 min each). They were then moved to 100% histoclear for 20 min at room temperature, washed twice with 50:50 histoclear/paraffin mix for 10 min at 65°C and then embedded in 100% paraffin. Paraffin was replaced three times to ensure removal of histoclear. Sections were cut at 10 μm. The protocol was similar for E14.5 fetuses except for longer dehydration steps (40 min each), followed by overnight storage in 100% ethanol. In this case, histoclear was added for 40 min at room temperature. Sections were either antibody stained (see immunofluorescence) or stained with Haematoxylin and Eosin. For bone staining, P1 pups were fixed overnight in 100% ethanol followed by three days in 100% acetone. They were then stained with Dawson staining solution overnight at 37°C, washed and cleared with 1% KOH at 37°C. Slower clearing was achieved with 10% glycerol in 1% KOH at room temperature. For Xgal staining, embryos were removed from culture and fixed in 2% PFA for 10 min, rinsed in 2 mM MgCl_2_ in PBS and incubated in staining solution: 1× PBS, 20 mM K3Fe(CN)6, 20 mM K4Fe(CN)6-3H2O, 2 mM MgCl_2_, 0.01% sodium deoxycholate and 0.02% NP-40 with X-gal (1 mg/ml final concentration) as a 1:50 addition at 37°C for 3 h.

### *In situ* hybridisation

*In situ* hybridisation of whole E10.5 embryos was performed essentially as previously described (Ybot-Gonzalez et al., 2005) using a cDNA probe for *Fgf8* (Crossley and Martin, 1995; variant 4, insert 800 bp) cloned into a pBluescript SK(+) vector. Sense and antisense riboprobes were generated using a digoxygenin RNA-labelling kit and T3/T7 RNA polymerases (Roche). Images are representative of at least three embryos per condition.

### Statistical analysis

Statistical analysis was performed in OriginPro 2017 (Origin Labs). Individual embryos were the unit of measurement. Comparison of two groups was carried out using an unpaired Student's *t*-test. Proportions were compared using Fisher's exact tests. Comparisons of slopes were based on Pearson's correlation. Graphs were made in OriginPro 2017 and are shown as dot plots or scatter plots. Analysis of Closure 5 presence was carried out blinded to genotype/treatment group, although marked PNP dysmorphology could not be masked in these analyses.

## Supplementary Material

10.1242/develop.202139_sup1 Supplementary information
