# Supplementary material for: Caudal Fgfr1 disruption produces localised spinal mis-patterning and a terminal myelocystocele-like phenotype in mice
Source: Development. 2023 Oct 10;150(19):dev202139. doi: 10.1242/dev.202139 (PMC10617625; doi:10.1242/dev.202139)
Supplement: Supplementary information [file develop-150-202139-s1.pdf]

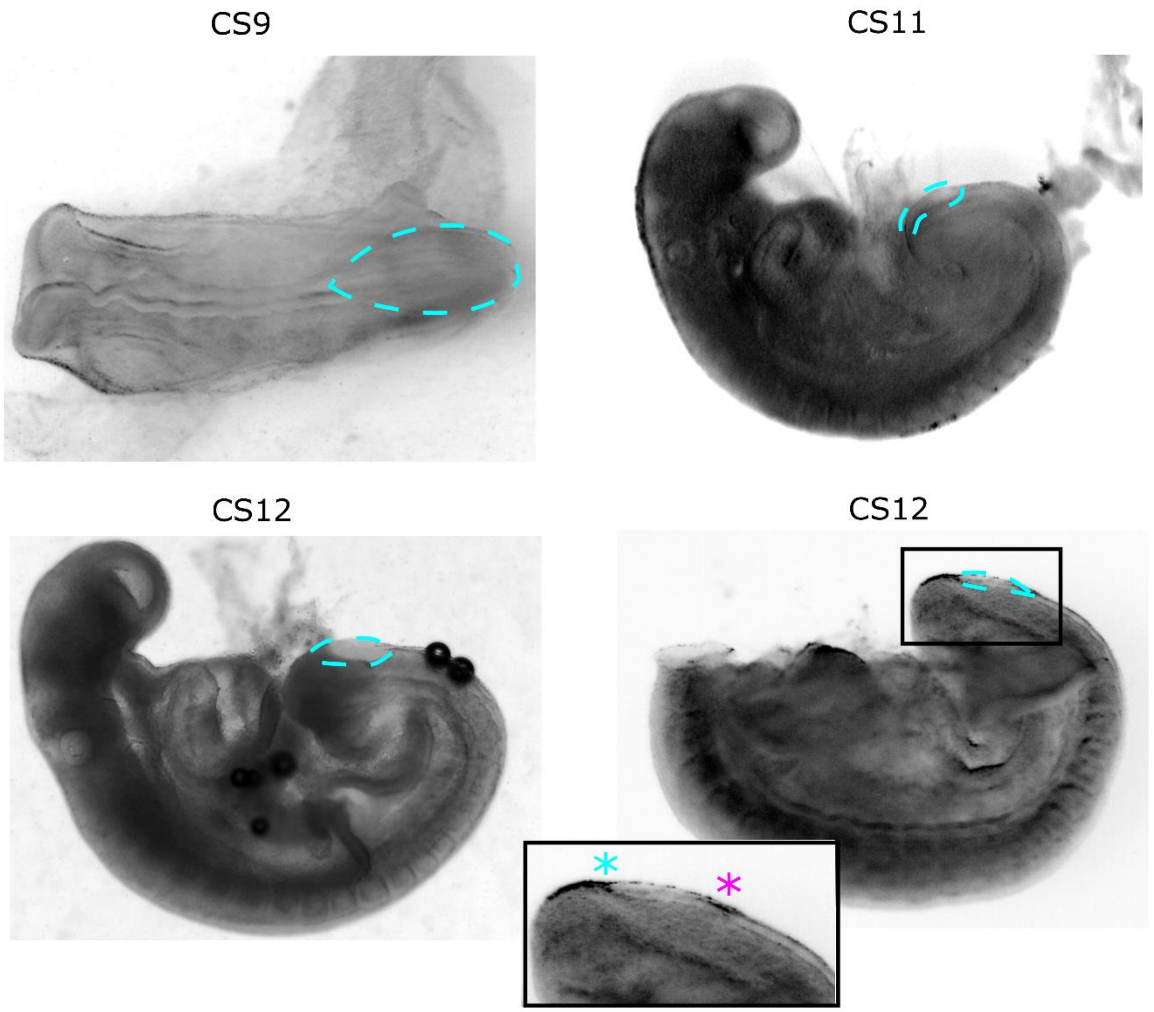

Fig. S1. Human PNPs have an elliptical morphology suggestive of Closure 5 formation at late stages of closure.

Brightfield images of human embryos at the Carnegie Stages (CS) indicated. The dashed cyan line annotates the posterior neuropore, which at CS9-11 has a spade-like morphology. By CS12, the human PNP has acquired an elliptical shape and the anatomy of the caudal extremity suggests formation of Closure 5 (cyan asterisk). The magenta asterisk indicates the rostral-to-caudal zippering point.

Development • Supplementary information

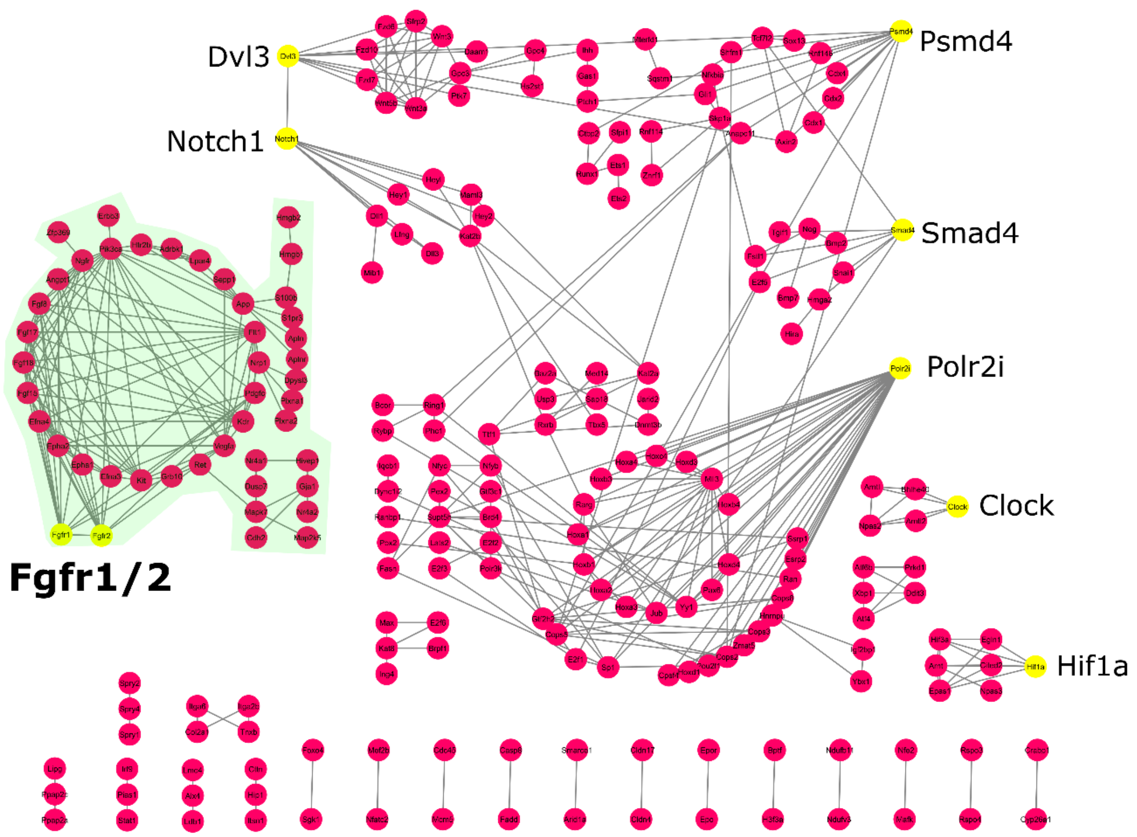

Fig. S2. FGF pathway components are known to be robustly expressed in the tissue region where Closure 5 forms.

523 genes known to be expressed in the region of Closure 5 formation (tail bud) between E9.5-E10.5 in mouse embryos were identified using the EMAGE gene expression database (http://www.emouseatlas.org/emage/)[1]. Genes whose protein products are known to interact with high confidence in reported experiments and curated databases were identified in StringDB[2]; non-interacting members were suppressed. The resulting network of interacting Closure 5-region genes was analyzed in Cytoscape[3] to identify sub-networks linked to nodes of interest (yellow, gene names annotated). Genes interacting with FGFR1 and 2 are indicated with green shading. Yellow nodes indicate genes related to specific pathway of interest. Results were obtained from bioinformatic analysis.

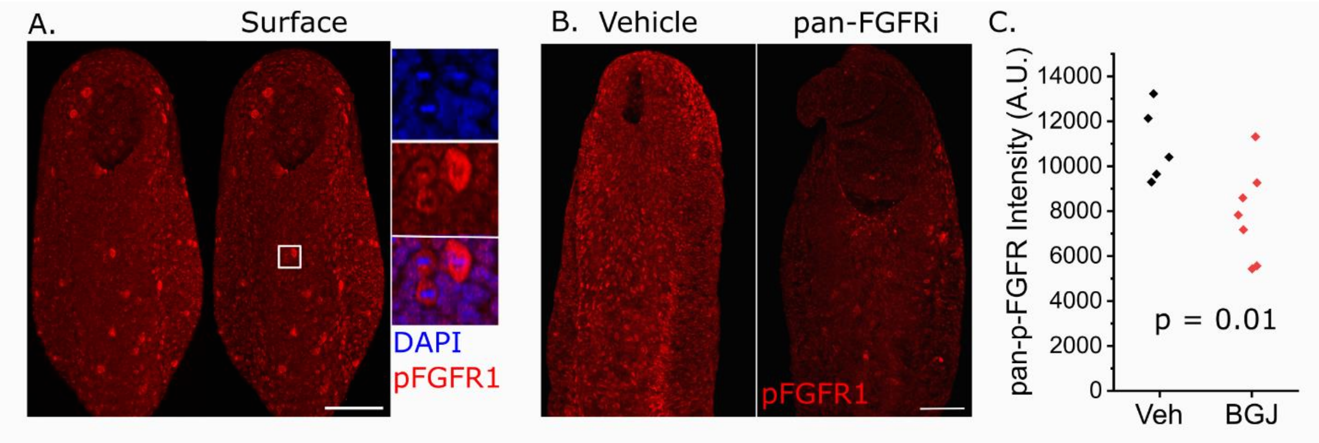

Fig. S3. Diminished p-FGFR1 following pharmacological antagonism.

A. Wholemount confocal image of a 28-somite embryo showing pFGFR1 immunolocalisation. The right panel is 'surface subtracted' to only show a 10 μm top surface, namely the surface ectoderm and apical neuroepithelium. Insert shows bright pFGFR1 labelling mitotic cells.

B. 'Surface subtracted' wholemount confocal images of embryos cultured in vehicle or pan-FGFR inhibitor for 24 hours. Scale bar = 100 μm.

C. pFGFR1 immunofluorescence intensity quantification in the 'surface subtracted' surface ectoderm of control and pan-FGFR inhibitor (BGJ) treated embryos. Surface subtraction was used to ensure the volume analysed is comparable between Z-stacks. P value by t-test.

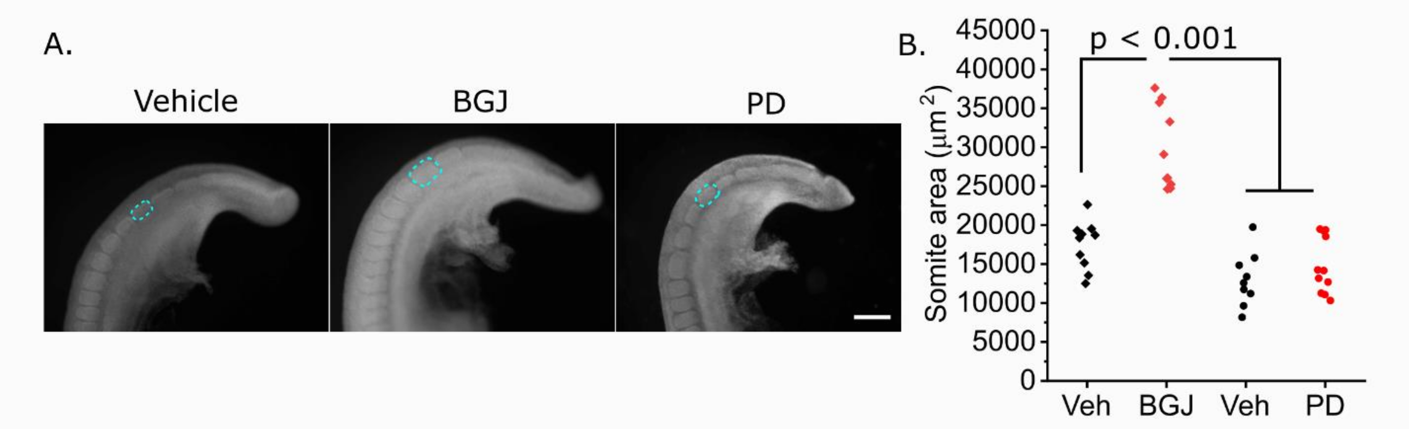

Fig. S4. Pan-FGFR inhibition increases somite size.

A. Fluorescent stereoscope images of DAPI-stained embryos treated for 24 hours with vehicle (DMSO), pan-FGFR inhibitor (BGJ) or FGFR1-targeting inhibitor (PD). Dashed cyan outlines indicate the penultimate somite. Scale bar = 225 μm.

B. Quantification of the projected area of the penultimate somite in FGFR-inhibited or vehicle-treated controls. The penultimate somite was chosen as this is more reliably visualized. Independent vehicle-treated littermate controls were included for both BGJ and PD cultures. Each point represents an embryo, p value by ANOVA with post-hoc Bonferroni.

Development • Supplementary information

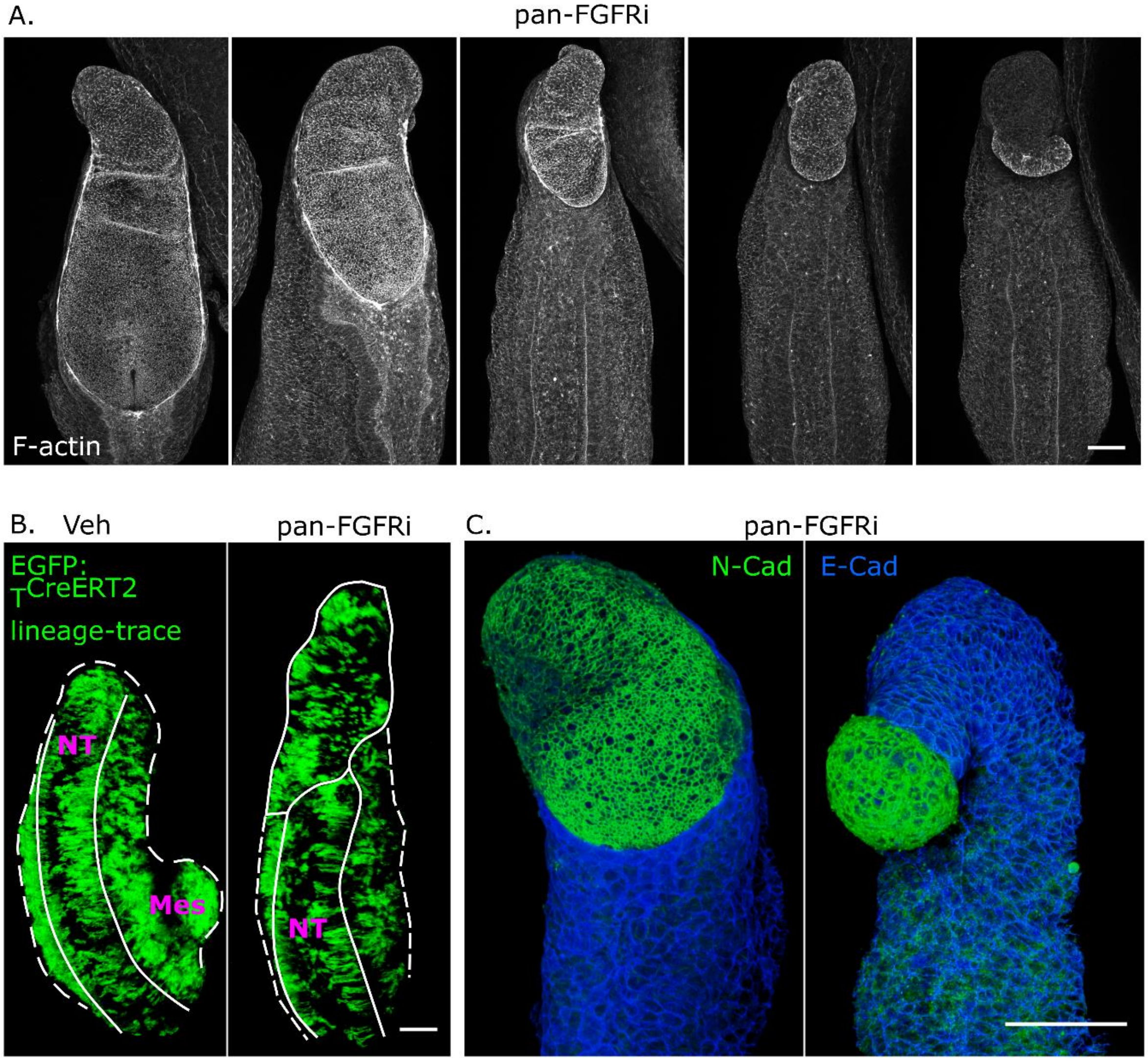

Fig. S5. Pharmacological inhibition of FGF signalling in cultured mouse embryos causes abnormal PNP closure.

A. Confocal images of phalloidin stained embryos cultured with pan-FGFR inhibitor for 24 hours.

B. 3D reconstructions of confocal-imaged vehicle and a pan-FGFR inhibitor treated embryo after 24 hours whole embryo culture. Neuromesodermal progenitors are lineage traced with $T^{CreERT2}$ and continue to give rise to both neural tube (NT) and mesoderm (Mes) in both conditions.

C. 3D reconstructions of confocal-imaged embryos after 24 hour culture with pan-FGFR inhibitor. Immunolocalisation of E- and N-cadherin shows that the PNP overgrowth is neuroepithelial.

Scale bars = 100 µm.

Development • Supplementary information

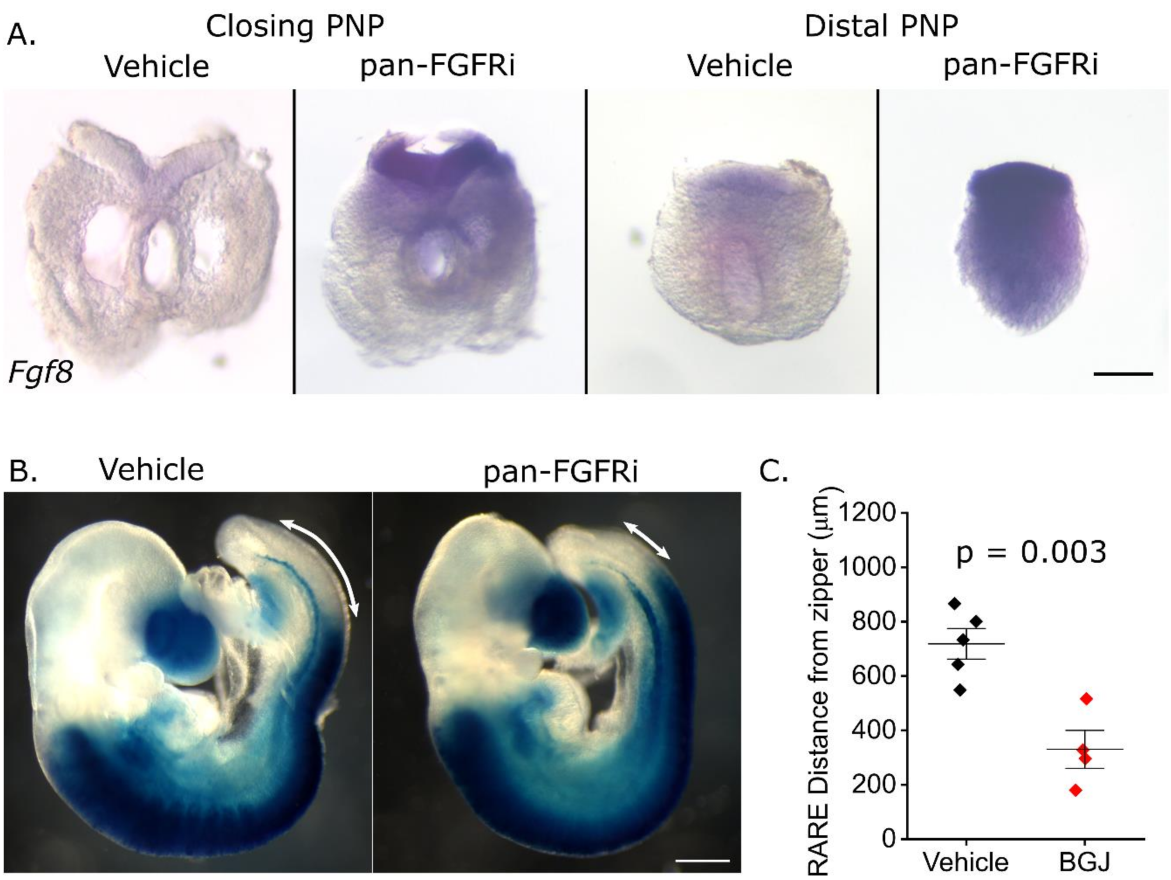

Fig. S6. Pan-FGFR inhibition for 8 hours increases *Fgf8* and causes caudal expansion of RARE signalling.

A. Vibratome sections of wholemount *Fgf8* in situ hybridisation in vehicle- and pan-FGFRi-treated embryos after 8 hours of whole embryo culture. Scale bar = 100 µm.
B. Brightfield images showing RARE-mediated LacZ expression domain in vehicle and pan-FGFRi-treated embryos after 24 hours of whole embryo culture. The double-headed white arrow indicates the distance between the RARE domain and the zippering point. Scale bar = 350 µm.
C. Quantification of RARE domain distance to the end of the zippering point in vehicle and pan-FGFRi (BGJ)-treated embryos after 8 hours of whole embryo culture. Points represent individual embryos.

Development • Supplementary information

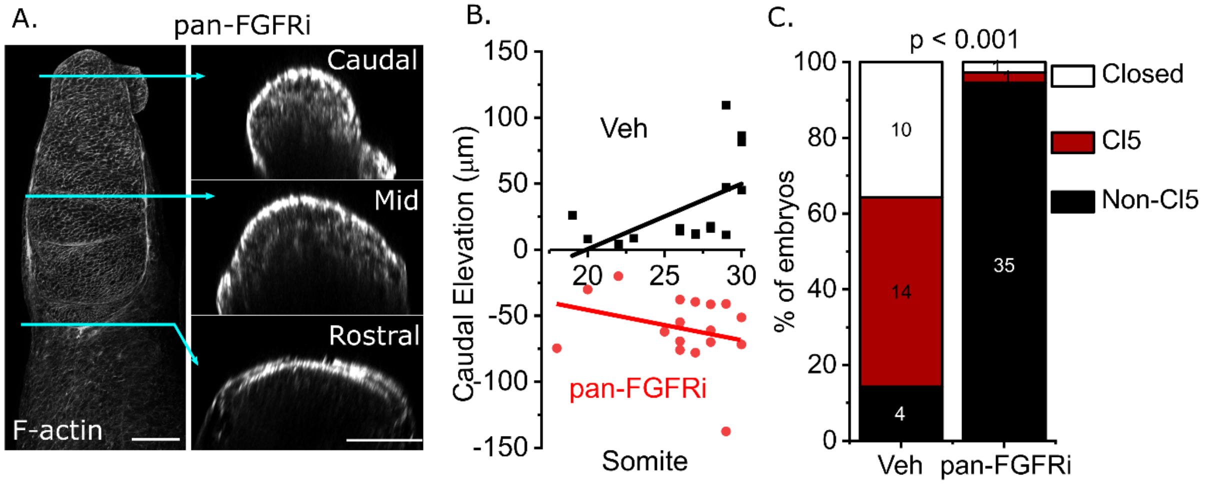

Fig. S7. Pharmacological inhibition of FGF signaling in cultured mouse embryos prevents neural fold elevation required for Closure 5 formation.

A. Confocal image of a phalloidin stained embryo after 24 hours culture with pan-FGFR inhibitor. The cyan arrows indicate the level of the optical cross-sections at the rostral, mid and caudal level. Scale bars = 100 µm.
B. Quantification of elevation at the caudal neural folds (90% of the PNP's length) in embryos at the indicated somite stages.
C. Quantification of the proportion of embryos collected at E10.5 with closed PNPs, open PNPs with Closure 5 morphology or open PNPs without Closure 5. Numbers indicate the number of embryos observed in each category.

Development • Supplementary information

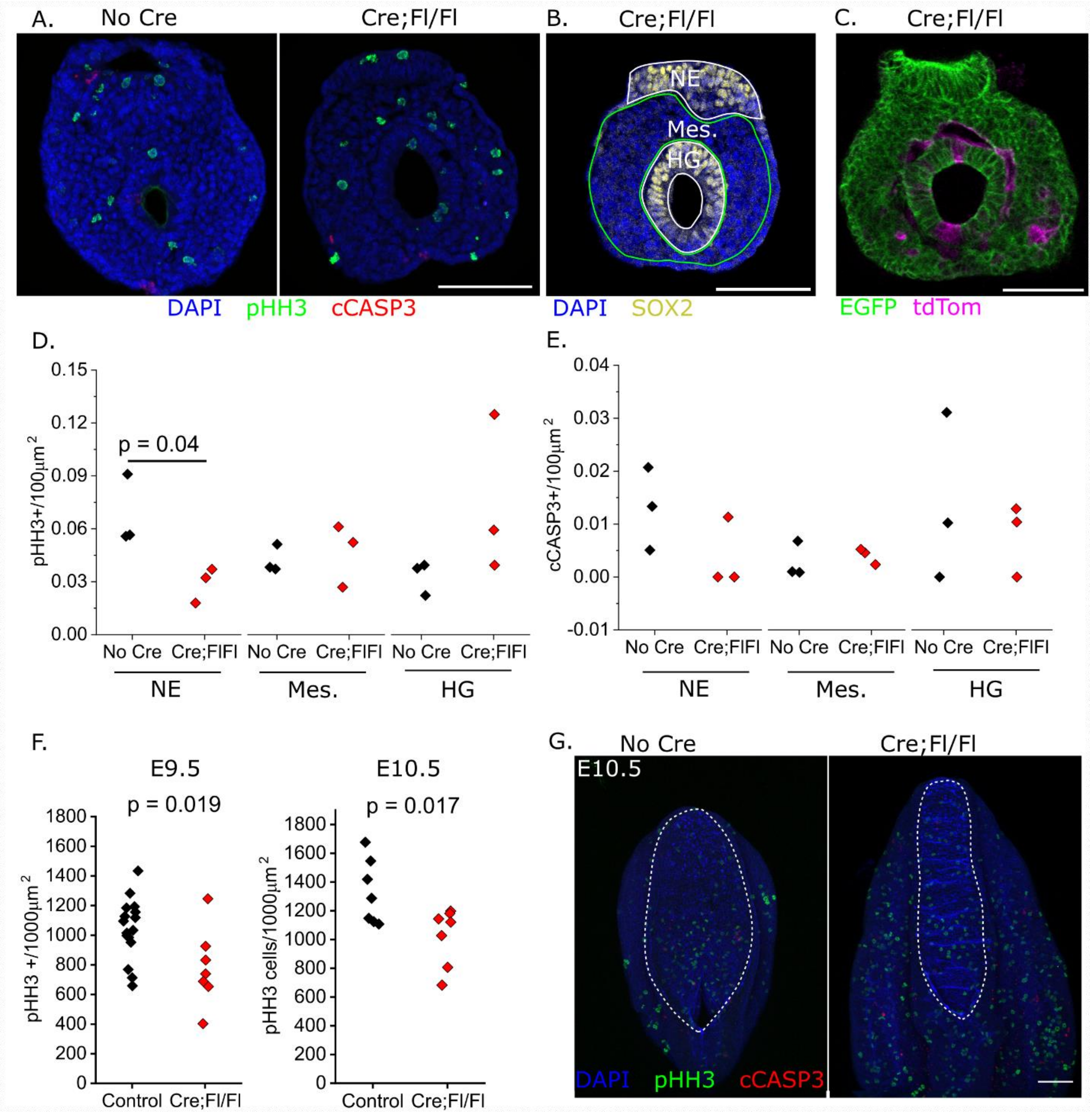

Fig. S8. *Fgfr1* deletion diminishes neuroepithelial proliferation.

A-B. Histological sections through the distal PNP of embryos at E10.5, when control embryos form Closure 5, immunofluorescently labelled to visualise the mitotic cell marker pHH3 and apoptosis marker cleaved caspase 3 (cCASP3). **B** shows annotation of the neuroepithelium (NE), mesoderm (Mes.) and hind-gut (HG) in an *Fgfr1*-deleted embryo immunolabelled for SOX2. Scale bars = 100 µm.

C. mTmG lineage tracing showing *Cdx2*^{Cre}-recombined (EGFP) and unrecombined (tdTom) cells in the distal tailbud of an E10.5 Cre;Fl/Fl embryo illustrating extensive lineage-tracing of most cells in this tissue. Scale bar = 100 µm.

Development • Supplementary information

D-E. Quantification of the number of proliferating or apoptotic cells per area in the NE, mesoderm or HG. Points represent individual embryos (n = 3 embryos per genotype).

F. Quantification of the number of mitotic cells per area of exposed neuroepithelium in control and Fgfr1-deleted embryos collected at E9.5 or E10.5 (embryos with closed PNPs were excluded). Points represent individual embryos.

G. Wholemount immunofluorescent images showing proliferating and apoptotic cells in control and Fgfr1-deleted embryos at E10.5. The dashed white line indicates the exposed neuroepithelium. Scale bar = 100 µm.

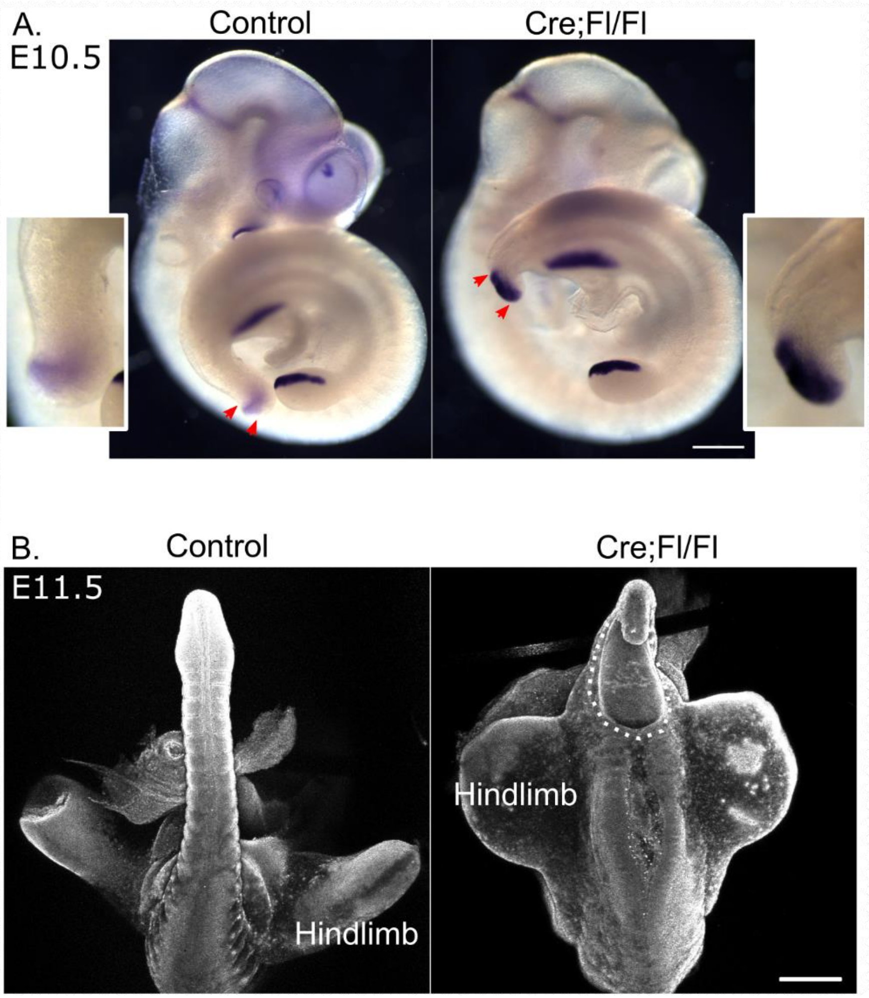

Fig. S9. *Fgf8* expression and late open neuropores in embryos with caudally-deleted *Fgfr1*.

A. Wholemount *Fgf8* in situ hybridisation in control and littermate Cre;Fl/Fl embryos. Arrowheads indicate the tailbud *Fgf8* domain, shown in the insets. Scale bar = 300 µm.

B. Reflection images of control and Cre;Fl/Fl littermates collected at E11.5. The dotted white line encircles the open lesion at the caudal end of the body axis. Scale bar = 500 µm.

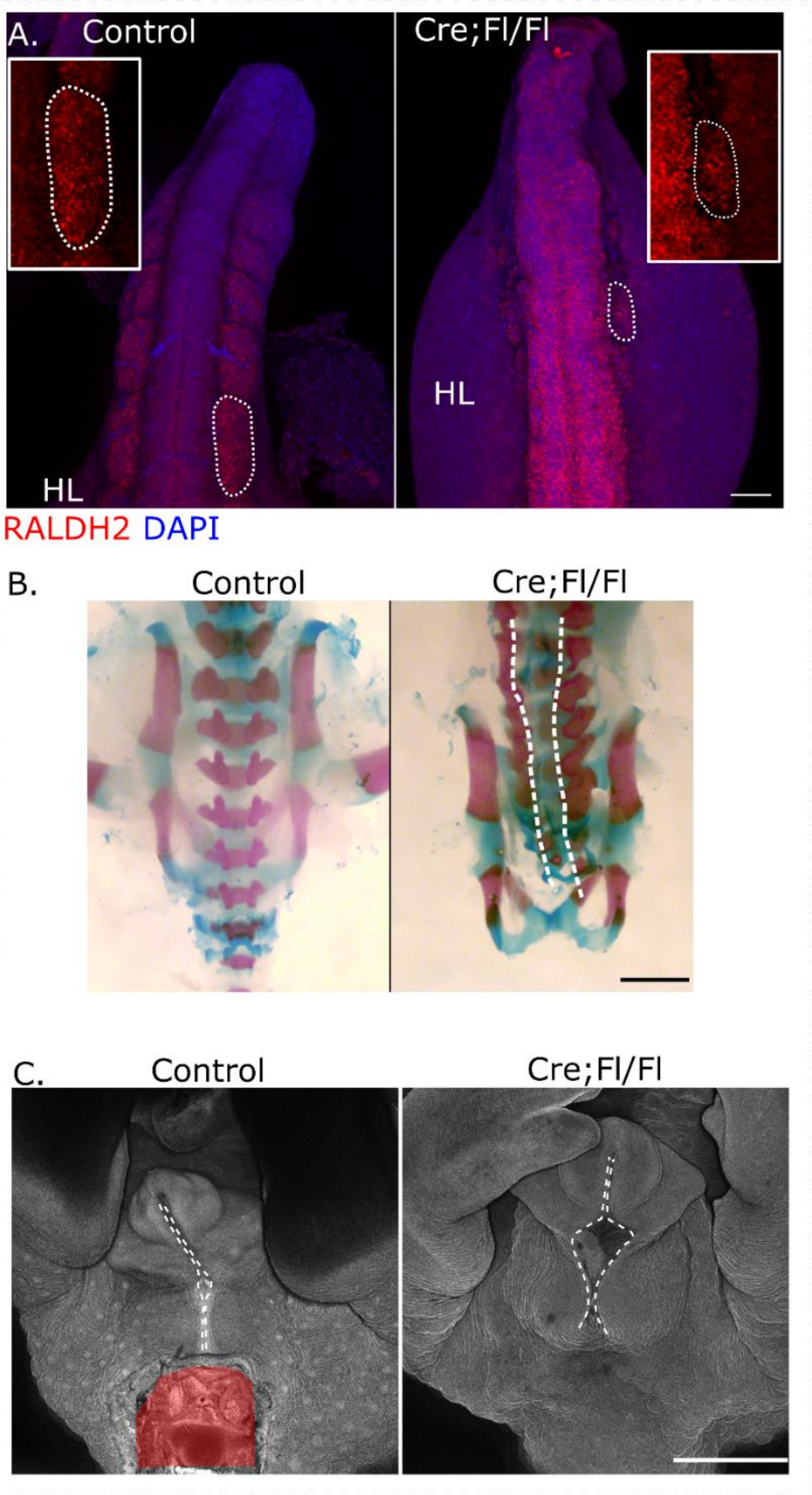

Development • Supplementary information

Fig. S10. Non-neuroepithelial abnormalities in embryos with caudally-deleted *Fgfr1*.

A. Wholemount immunofluorescent visualization of RALDH2 in an E10.5 control and littermate Cre;Fl/Fl embryo. Inserts show magnified views of a somite in each embryo (dashed lines). HL = hindlimbs, scale bar = 100 µm.

B. Whole-mount skeletal staining with Alcian blue and Alizarin red of control and Cre;Fl/Fl P1 pups from the same litter. Dashed white lines indicate bifid neural arches. Scale bar = 850 µm.

C. Reflection images of control and Cre;Fl/Fl littermates collected at E16.5. The dotted line annotates the perineum, which is fused in control and partially open in the mutant. Red area shows the tail base, which was cut to enable imaging of underlying structures in the control fetus. Scale bar = 1 mm.

Development • Supplementary information

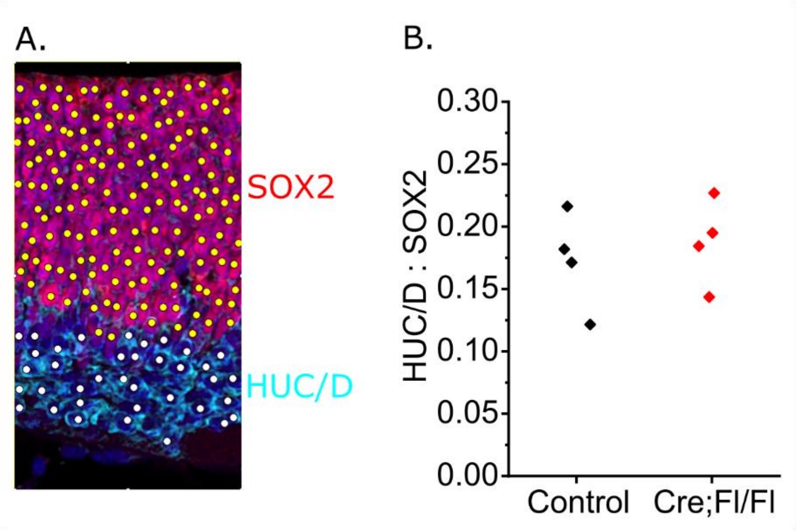

Fig. S11. *Fgfr1* disruption does not diminish neurogenesis in the distal spine.

A. Representative image of a 100 µm length of the lateral neural tube stained and imaged equivalently to embryos in Figure 5G. Yellow dots indicate SOX2-bright cells, white dots indicate HUC/D-bright cells.
B. Quantification of the ratio of HUC/D to SOX2 bright cells along 100 µm lengths of the lateral neural tube in control and *Fgfr1*-disrupted embryos. Points represent individual embryos.

Development • Supplementary information

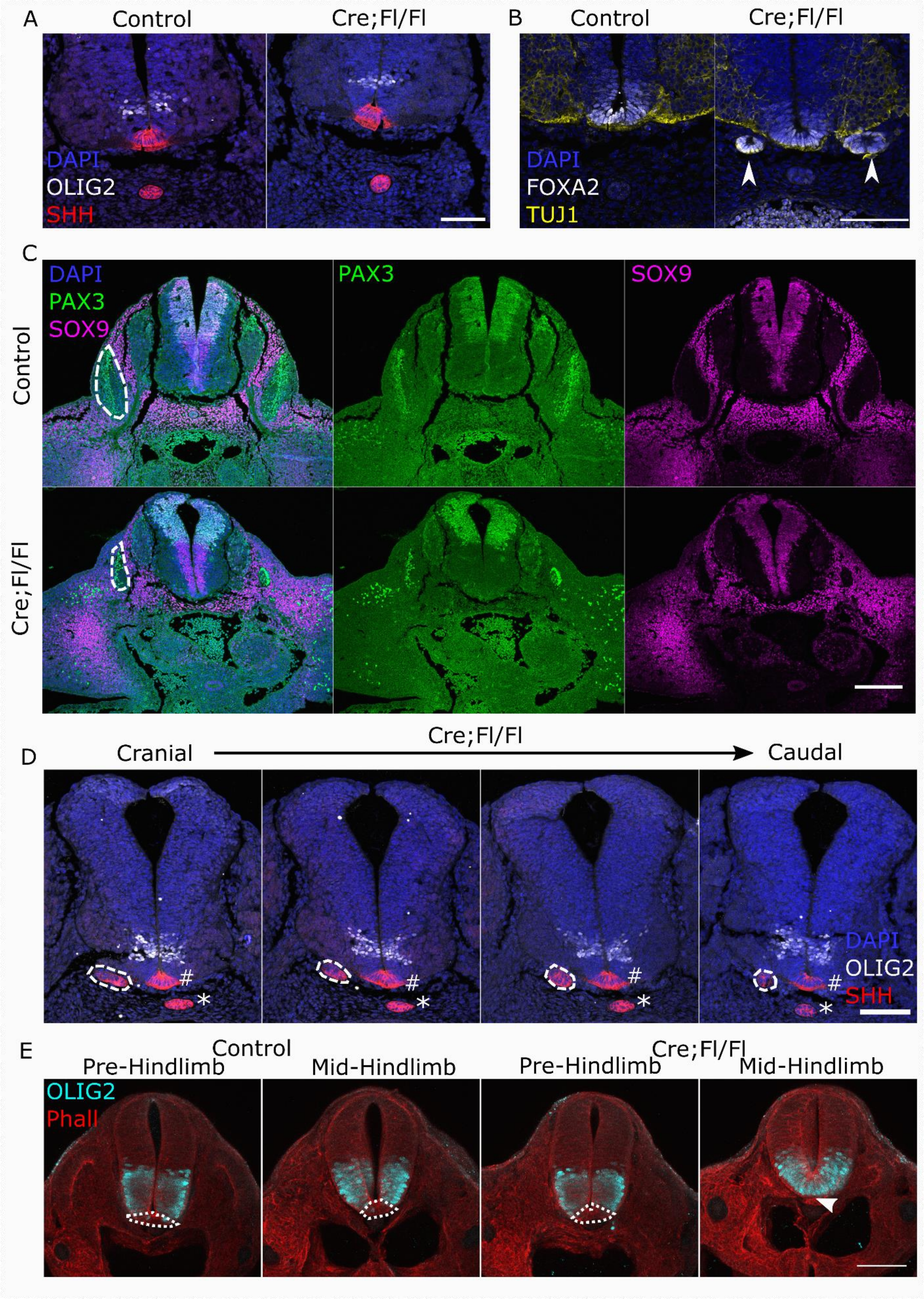
A
Control
Cre;Fl/Fl
DAPI
OLIG2
SHH
B
Control
Cre;Fl/Fl
DAPI
FOXA2
TUJ1
C
DAPI
PAX3
SOX9
PAX3
SOX9
Control
Cre;Fl/Fl
D
Cranial
Cre;Fl/Fl
Caudal
#
*
DAPI
OLIG2
SHH
E
Control
Pre-Hindlimb
Mid-Hindlimb
Cre;Fl/Fl
Pre-Hindlimb
Mid-Hindlimb
OLIG2
Phall

Fig. S12. Caudal *Fgfr1* deletion causes localized progenitor domain abnormalities.

A-C Immunofluorescent localization of neuronal progenitor markers through the lumbar spinal cord of control and Cre;Fl/Fl littermates collected at E11 showing: A) pMN marker OLIG2 and floor plate and notochord marker SHH. B) Post-mitotic neuronal marker Tuj1 and floor plate marker FOXA2. The arrowheads indicate ectopic FOXA2 foci in the Cre;Fl/Fl embryo. C) Dorsal/neural crest marker PAX3 and neuroepithelial/neural crest marker SOX9. The dashed line encircles the dermomyotome which is markedly reduced in the Cre;Fl/Fl embryo. Scale bars A,B = 100 µm, C = 200 µm.

D. Serial sections through the lumbar spinal cord of a Cre;Fl/Fl embryo collected at E11 and stained for OLIG2 and SHH. SHH stains the floor plate (#), notochord (*) and ventral ectopic clusters encircled by a dotted line. Scale bar = 100 µm.

E. Sections at pre- and mid-hindlimb level of control and Cre;Fl/Fl littermates collected at E10.5. The OLIG2 negative floor plate (dotted line), disappears at the mid-hindlimb level of the mutant (white arrowhead). Scale bar = 100 µm.

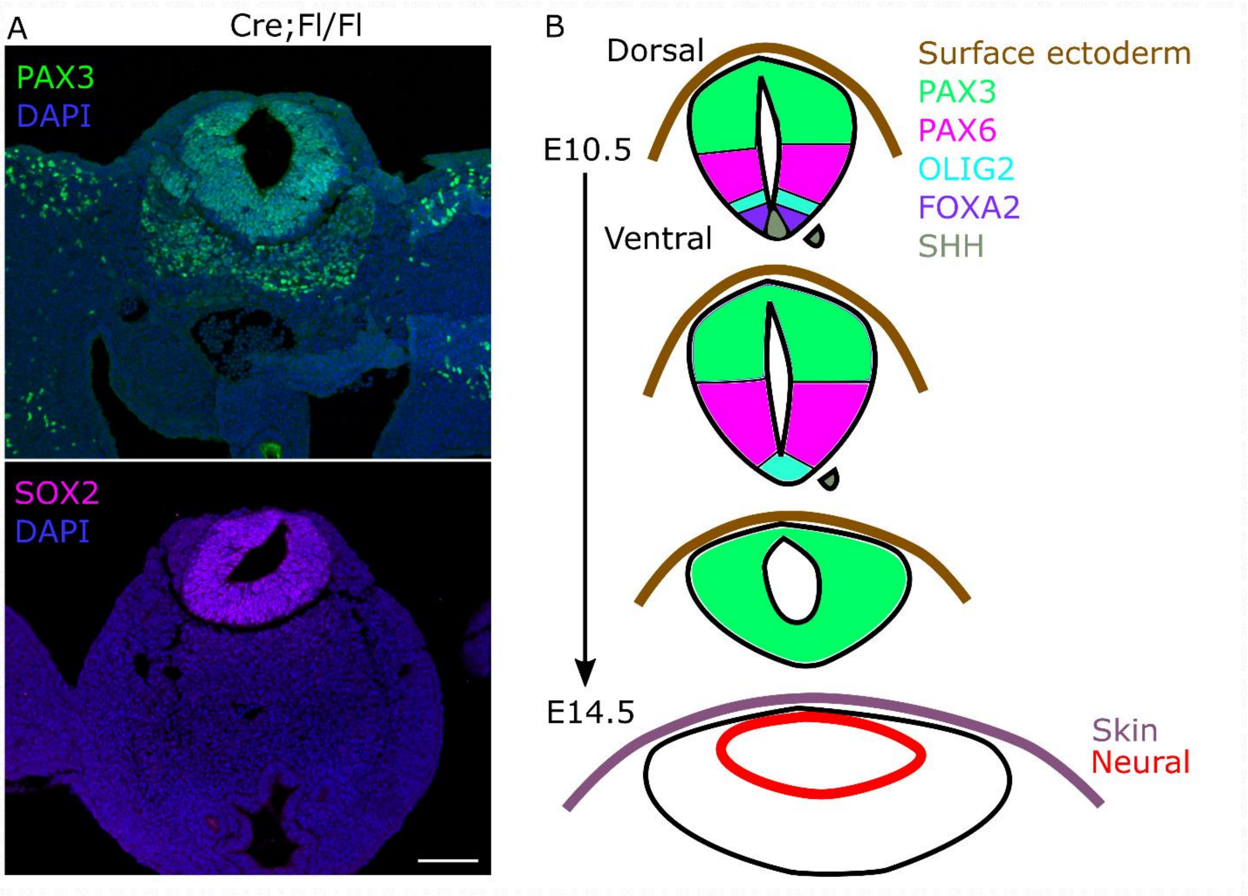

Fig. S13. Neural tube dorsalization precedes its dysmorphology and central canal dilation in *Fgfr1*-disrupted embryos.

A. Hindlimb level section through a Cre;Fl/Fl embryo collected at E11.5. The neural tube lumen is circular and stains positive for SOX2 and PAX3 throughout. Scale bar = 200 µm.

B. Schematic representation showing the progressive neural tube dorsalisation after caudal deletion of *Fgfr1* in Cre;Fl/Fl embryos. Ectopic clusters of SHH and FOXA2 at E10.5 precede loss of ventral progenitor domains. This leads to ventral expansion of PAX3 and PAX6 from E11.5, which finally encircle the neural tube lumen. Central canal dilation produces a terminal myelocystocele-like phenotype at E14.5.

Supplementary References

1 Richardson, L. *et al. Nucleic Acids Res* **42**, D835-844, doi:10.1093/nar/gkt1155 (2014).
2 von Mering, C. *et al. Nucleic Acids Res* **33**, D433-437, doi:10.1093/nar/gki005 (2005).
3 Shannon, P. *et al. Genome Res* **13**, 2498-2504, doi:10.1101/gr.1239303 (2003).

Development • Supplementary information
