# Supplementary material for: Caudal Fgfr1 disruption produces localised spinal mis-patterning and a terminal myelocystocele-like phenotype in mice
Source: Development. 2023 Oct 10;150(19):dev202139. doi: 10.1242/dev.202139 (PMC10617625; doi:10.1242/dev.202139)
Supplement: Supplementary file 1 [file develop-150-202139_review_history.pdf]

Caudal *Fgfr1* disruption produces localised spinal mis-patterning and a terminal myelocystocele-like phenotype in mice.

Eirini Maniou, Faduma Farah, Abigail R. Marshall, Zoe Crane-Smith, Andrea Krstevski, Athanasia Stathopoulou, Nicholas D.E. Greene, Andrew J. Copp and Gabriel L. Galea
DOI: 10.1242/dev.202139

Editor: Liz Robertson

Review timeline

Original submission:	23 March 2023
Editorial decision:	25 April 2023
First revision received:	29 June 2023
Editorial decision:	20 July 2023
Second revision received:	24 August 2023
Editorial decision:	7 September 2023
Third revision received:	9 September 2023
Accepted:	11 September 2023

Original submission

First decision letter

MS ID#: DEVELOP/2023/201808
MS TITLE: Caudal Fgfr1 disruption produces localised spinal mis-patterning and a terminal myelocystocele-like phenotype in mice
AUTHORS: Eirini Maniou, Faduma Farah, Zoe Crane-Smith, Andrea Krstevski, Athanasia Stathopoulou, Nicholas DE Greene, Andrew J Copp, and Gabriel L Galea
ARTICLE TYPE: Research Article

I have now received all the referees reports on the above manuscript, and have reached a decision. The referees' comments are appended below, or you can access them online: please go to BenchPress and click on the 'Manuscripts with Decisions' queue in the Author Area.

As you will see from their reports, the referees recognise the potential of your work, but they also raise significant concerns about it. The referees acknowledge that the study provides a useful description of closure 5 in mouse and human embryos, however the function data for the role of FGFRs isn't conclusive. The referees raise concerns about the discrepancies between the pharmacological inhibition and genetic deletion experiments and question the choice of Cre driver. The lack of clear mechanistic insight raises doubts about how well Fgfr1 signalling loss mimics the human condition. Given the nature of these concerns, I am afraid I have little choice other than to reject the paper at this stage.

However, having evaluated the paper, I do recognise the potential importance of this work. I would therefore be prepared to consider as a new submission an extension of this study that contains new experiments, data and discussions and that address fully the major concerns of the referees. Providing evidence for the cellular basis of the defects would greatly strengthen the study. However I recognise that this work required goes beyond a standard revision of the paper. Please bear in mind that the referees (who may be different from the present reviewers) will assess the novelty of your work in the context of all previous publications, including those published between now and the time of resubmission.

© 2023. Published by The Company of Biologists under the terms of the Creative Commons Attribution License (https://creativecommons.org/licenses/by/4.0/).

Reviewer 1

Advance summary and potential significance to field

This manuscript examines the effects of loss of Fgfr1 in posterior neural tube closure. The authors begin by drawing comparisons between murine and human early development to show that the neural tube closure point, closure 5, is an evolutionarily shared structure between both species. Next, they use pharmacological inhibition of FGF signaling on whole murine embryos and find that neural tube closure is delayed, with neural tube defects seen at closure 5. The authors then use the caudally expressed Cdx2Cre to conditionally delete Fgfr1 and find similar neural tube closure defects to those observed upon pharmacological inhibition. The authors also note that the shape of the neural tube is disrupted even after closure in conditional mutants, exhibiting signs of cystic dilation at the level of the hindlimbs. The authors find that the neural tube is abnormally patterned in conditional Fgfr1 mutants, as expression the dorsal neural tube marker Pax3 is expanded, resulting in loss of ventral identity. The authors conclude that taken together, these findings provide a model of terminal myelocystocele, a form of closed spina bifida, that helps elucidate how this type of neural tube defect arises.

Comments for the author

Major Concerns
1. As Fgfr1 has known roles in paraxial mesoderm development, which the authors note in the text, the fact that they do not see defects when using pharmacological inhibition could mean that penetration of the treatment may not be optimal in whole embryos. They do note that somitic mesoderm is hypomorphic in the conditional mutants, which is more in line with previously published data. The authors might consider discussing these discrepancies in more detail in the discussion, in the results, or where relevant.
2. The authors should more clearly state their intentions behind using the Cdx2Cre for the conditional deletion. It is great that they used the mTmG reporter to confirm that recombination was occurring in the regions that were affected, but the broadness of the Cre expression (which is expressed in more than just the neural tube) muddies the results. The authors briefly address the idea of crosstalk between cell types in the discussion, but autonomous vs. non-autonomous effects of FGF signaling on the neural tube defects cannot be established, calling into question the relevance of the resulting phenotype. While utilizing another Cre line would drastically increase the timeline and might unreasonable, the authors should at least discuss other Cre lines that might be relevant but explain why they were not used.
3. The authors state that PD173074 is an Fgfr1 specific inhibitor, however there is evidence that PD173074 can also inhibit the other Fgfrs as well (see PMID 15116089). As such, the similar phenotypes seen between BGJ398 and PD173074 is not that surprising.
4. Additionally, both inhibitors have listed minor effects on VEGFR2 and Src family kinases. While the effects are listed as several fold lower, per the manufacturers, small disruptions may have effects on the embryo as the treatment was over 24hrs. These potential off-target effects could be another explanation for the discrepancy in the phenotypes observed in the pharmacologically treated embryos versus the conditional deletion embryos. These limitations should be discussed.
5. The authors note that pERK1/2 persists on the PNP rim even after treating with BGJ398. While FGFRs are a major driver of ERK1/2 activity, there are numerous other RTKs that can drive ERK1/2 activity as well. The presence or absence of pERK1/2 therefore should not be used as sole evidence of FGFR activity or lack thereof. Based on efficacy in controls, perhaps an anti-pFGFR antibody could be used to verify the reduced activity of FGFRs in pharmacologically treated embryos.

Minor Concerns
1. Similar neural tube closure defects have been reported in Fgfr1 mutants previously (PMID 317047; 16421190; 4573858), although not thoroughly explored, reducing the novelty of these findings somewhat. The authors should cite these papers.
2. The title of figure 2 "Tissue-specific functions of FGF signaling, likely through FGFR1, promote timely completion of PNP closure" instills a sense of vagueness. The statement should be clarified to be less ambiguous.
3. The manuscript should be read through for typos, as a number are present:

© 2023. Published by The Company of Biologists under the terms of the Creative Commons Attribution License (https://creativecommons.org/licenses/by/4.0/).

a. Abstract line 2: classed -> classified
b. Into para 2 line 3: primary -> primarily
c. Results para 7 line 4-6: numbers are duplicated
d. Results para 9 line 1: as -> a
e. Gene and protein names should be consistent for the species, as many mouse genes are not italicized properly, and clarification should be made as to whether a protein or gene is being discussed via proper nomenclature.

Reviewer 2

Advance summary and potential significance to field

This paper develops the authors previous work identifying caudal neurulation closure point (5) in mouse embryos, characterising similar conserved morphological changes in human embryos. Defects in neurulation at this site may underlie terminal myelocystocele and the authors set out to develop a mouse model in which to investigate mechanisms regulating this caudal closure event. This takes place in a region characterised by Fibroblast Growth Factor (FGF) signalling and the authors show that attenuation of this pathway, using small molecule or conditional genetic deletion approaches, generates delayed neural tube closure and a phenotype resembling terminal myelocystocele. Careful histological analysis and examination of expression patterns of cell type specific markers further reveal a complex phenotype: with delayed neural tube closure, formation of ectopic neural cell clusters as well as progressive loss of expression of ventral spinal cord progenitor markers, including eventual Olig2 loss and predominant expression of the dorsal marker Pax3, indicating spinal mis-patterning even after neural tube closure following attenuation of Fgfr signalling.

Overall, the paper presents useful largely descriptive data that align mouse and human neural tube Closure 5 and uncovers a complex phenotype following FGFR loss. This lays the foundation for further mechanistic studies on how this closure event is regulated, and how associated myelocystocele formation results from its perturbation. While it is not clear how well Fgfr1 signalling loss mimics the human condition, the work does support the possibility that this closure event is vulnerable to perturbation of Fgfr and related pathways.

Comments for the author

This is an interesting study which provides a careful description of closure 5 in mouse and human embryos. The main data of the paper involves experiments attenuating FGFR signalling using either small molecule inhibitor or conditional genetic approach. These reveal complex phenotypes that while implicating FGFR signalling in this closure event also indicate multiple downstream events, for example, ectopic, mis-localised Sonic hedgehog signalling, which likely contribute to the generation of a myelocystocele-like morphology. There are also concerns about the precision of some experiments, which may contribute to complex phenotypes described here. Some specific points the authors may wish to consider for future revision include:

i) Experiments exposing embryos to the Pan FGFRi BGJ 398 (or PD 173074 mentioned in the Methods) for 24h appear not to have included assessment of the period when the inhibitor is active - data from cells in vitro suggests it is possible that this was for only 3-6h. Analysis of embryos in these experiments at 24h may then reflect phenotypes due to acute impact on morphological events, such as neurulation, while lack of apparent effect on other tissues such as somites is due to only transient loss of FGFR signalling? Along the same lines, it is difficult to interpret the dpERK detection at 24h in Fig2A, does this reflect action of resumed FGFR signalling, other RTK signalling pathways, or another ERK activating mechanism?

© 2023. Published by The Company of Biologists under the terms of the Creative Commons Attribution License (https://creativecommons.org/licenses/by/4.0/).

ii) Given that FGFR signalling promotes proliferation and in many contexts protects against cell death it would be informative to determine whether these fundamental processes, (which would affect tissue available for neurulation or for adjacent non-neural tissue providing biomechanical support) were affected following attenuation of this pathway. In fig2C the pan FGFRi embryo appears to lack caudal tissue. It is not clear from fig2B whether this is the case in this embryo assessed for Fgf8 expression. Sections of such embryos and some cell counts would be informative.

iii) Figure 5D - ideally cell counts for neurons rather area in which TUJ1 expression is found is needed to conclude reduced neuron numbers. This can be done using a marker that localised to the cell body, for example HuC/D. For the TUJ1 data provided the numbers of sections and embryos sampled should be provided - I could not find this information in any of the figure legends nor in Methods, this should be provided for all quantifications in the paper - ideally in the figure legends.

The discussion is wide ranging and raises further potential changes that may contribute to the complex phenotype that emerged from conditional Fgfr1 caudal loss. While these possibilities might be investigated to dissect distinct aspects of this FGFR signalling perturbation phenotype (e.g. ectopic Shh), this would require extensive further focussed investigation, which seems beyond the scope of this initial report and may not reveal a primary, initiating cause of this complex neurulation defect. The lack of clear mechanistic insight here leaves this a primarily descriptive study with no clear next steps for further insights. The authors have previously described closure 5 and this appears a largely incremental set of analyses, which while implicating FGFR1 also show that it is all much more complex.

Reviewer 3

Advance summary and potential significance to field

Caudal Fgfr1 disruption produces localised spinal mis-patterning and a terminal myelocystocele-like phenotype in mice Maniou et al.

The manuscript provides evidence that a posterior neuropore (PNP) closure point 5 that these investigators previously described in the mouse embryo also exists in the human embryo, demonstrates in Carnegie stage 11 (CS11) human embryos. Further pursuing the mouse model, investigators show data supporting the role of FGF signaling, and Fgfr1 in particular, in the successful closure of the caudal end of the spinal cord. Either pharmacological blockade of Fgfr or conditional deletion of Fgfr1 impairs closure 5 formation and distorts the PNP, which nonetheless achieves closure. Post closure, the distal neural tube re-opens to create a flared central canal between hindlimbs to form a distal fluid-filled sac resembling a terminal myelocystocele. Spinal progenitor domains are distorted so that there is a progressive loss of ventral progenitor domains preceding cystic dilatation. Authors conclude that a terminal myelocystocele-like phenotype can arise post NT closure, attributable to localized spinal mis-patterning in the absence of Fgfr1 signaling.

This is a significant paper that addresses the mechanism leading to caudal myelocystocele at the junction between primary and secondary NT closure in vertebrate embryos, including human. While the interplay of Pax3, FGF signaling opposition to somitic mesoderm-derived retinoic acid, repressed Shh, Sox2 and ventral Olig2 have all been implicated in the formation of the terminal spinal cord, more precise definition of the events has been lacking, since global knockout of Fgfr1 causes embryonic lethality soon after gastrulation. A Cdx2Cre driver was used to inactivate Fgfr1 to generate embryos with an open PNP at E10.5. Nevertheless, embryos were able to ultimately achieve complete closure, especially unexpected since other disruptions of Fgfr1 (chimeras or exon 3 deletion of the Fgfr1a isoform) can lead to open spina bifida in a proportion of embryos.

Thus, an interesting observation in this model is the apparent continuum of open to closed spina bifida, suggesting shared genetic basis. Authors propose that whereas failed neurulation leads to open myelomeningocele, neural patterning defects contribute to various closed NTDs.

© 2023. Published by The Company of Biologists under the terms of the Creative Commons Attribution License (https://creativecommons.org/licenses/by/4.0/).

The authors' rationale and interpretations are well described. The histology figures are beautifully prepared and statistical data are clear. The combination of pharmacological and conditional inactivation models is compelling.

I would only recommend that more information be provided in methods regarding the "Human Developmental Biology Resource tissue bank" with a link to their website. This will provide the reader with information relating to human subjects protections and ethical considerations.

Comments for the author

I do not see that major revisions are needed. It is highly suitable for publication. My suggestion is to include more information regarding the source of human embryo material.

First revision

Author response to reviewers' comments

We thank the reviewers for their careful reading of our manuscript and constructive comments which have helped us improve it. We have undertaken additional experiments and provide new data which fully address their comments and support our original conclusions. The revised manuscript now has six main figures and 13 supplementary figures.

Reviewer 1 Comments for the Author:
Major Concerns
1. As Fgfr1 has known roles in paraxial mesoderm development, which the authors note in the text, the fact that they do not see defects when using pharmacological inhibition could mean that penetration of the treatment may not be optimal in whole embryos. They do note that somitic mesoderm is hypomorphic in the conditional mutants, which is more in line with previously published data. The authors might consider discussing these discrepancies in more detail in the discussion, in the results, or where relevant.

In the original submission we had counted the number of somites in cultured embryos, but had not analysed their morphology. We have now performed additional experiments to analyse the size of the penultimate somite, finding that pan-FGFR inhibition increases somite size. This is consistent with a previous study in chick embryos from the Pourquie lab (Dubrulle et al Cell 2001), in which a different FGFR antagonist was reported to increase the number of cells allocated to each somite. We did not observe a change in somite size when using the second, FGFR1-targeting antagonist despite causing equivalent PNP phenotypes. This data is now shown in a new figure, Supplementary Figure 4 (below).

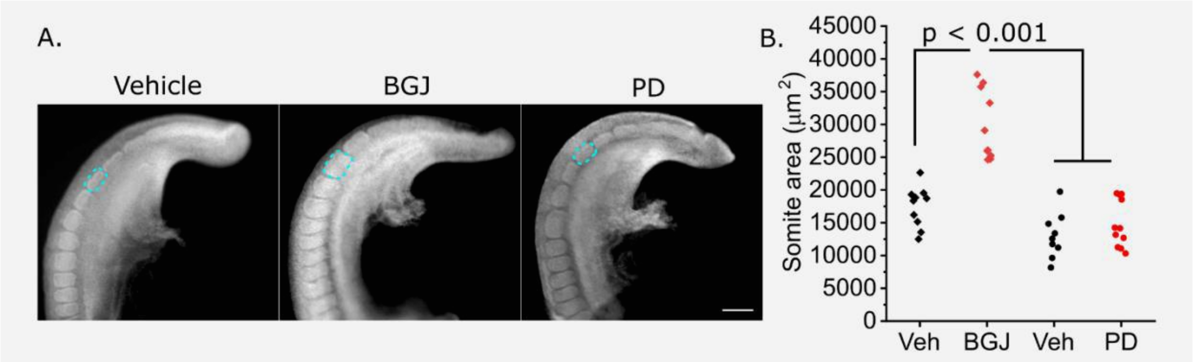

Supplementary Figure 4: Pan-FGFR inhibition increases somite size.

A. *Fluorescent stereoscope images DAPI-stained embryos treated for 24 hours with vehicle (DMSO), pan-FGFR inhibitor (BGJ) or FGFR1-targeting inhibitor (PD). Dashed cyan outlines indicate the penultimate somite. Scale bar = 225 μm.*

© 2023. Published by The Company of Biologists under the terms of the Creative Commons Attribution License (https://creativecommons.org/licenses/by/4.0/).

B. Quantification of the projected area of the penultimate somite in FGFR-inhibited or vehicle-treated controls. The penultimate somite was chosen as this is more reliably visualized. Independent vehicle-treated littermate controls were included for both BGJ and PD cultures. Each point represents an embryo, p value by ANOVA with post-hoc Bonferroni.

2. The authors should more clearly state their intentions behind using the Cdx2Cre for the conditional deletion. It is great that they used the mTmG reporter to confirm that recombination was occurring in the regions that were affected, but the broadness of the Cre expression (which is expressed in more than just the neural tube) muddies the results. The authors briefly address the idea of crosstalk between cell types in the discussion, but autonomous vs. non-autonomous effects of FGF signaling on the neural tube defects cannot be established, calling into question the relevance of the resulting phenotype. While utilizing another Cre line would drastically increase the timeline and might unreasonable, the authors should at least discuss other Cre lines that might be relevant but explain why they were not used.

The Cre line used was rationally chosen because of its expression domain and timing. No other Cre driver, of which we are aware, lineage traces all PNP neuroepithelial cells before Closure 5 formation without recombining in other cell types. We have now added discussion of our Cre-driver selection, including expanding reference to previous studies using different Cre drivers which did not report equivalent neural tube phenotypes:

"Identifying the specific cell types in which *Fgfr1* signalling promotes neural fold elevation will require future tissue-specific deletion studies. Previous studies which deleted *Fgfr1* using *T^{Cre}* reported musculoskeletal phenotypes comparable to those observed here, but not PNP dysmorphology or later terminal myelocystocele-like phenotypes (32, 46). Similarly, *Pax3^{Cre}* deletion of *Fgfr1* caused kidney malformation, but neural tube defects were not reported (47). It is not clear whether neural tube phenotypes were carefully assessed in those studies. *Cdx2^{Cre}* used in this study provides near-global gene deletion in the caudal embryo which starts sufficiently late in development to avoid lethality, yet sufficiently early to impact completion of neural tube closure."

3. The authors state that PD173074 is an Fgfr1 specific inhibitor, however there is evidence that PD173074 can also inhibit the other Fgfrs as well (see PMID 15116089). As such, the similar phenotypes seen between BGJ398 and PD173074 is not that surprising.

The new somite size data provided above shows these two compounds are not redundant. Ultimately, our claim of FGFR1 involvement is based on genetic deletion.

4. Additionally, both inhibitors have listed minor effects on VEGFR2 and Src family kinases. While the effects are listed as several fold lower, per the manufacturers, small disruptions may have effects on the embryo as the treatment was over 24hrs. These potential off-target effects could be another explanation for the discrepancy in the phenotypes observed in the pharmacologically treated embryos versus the conditional deletion embryos. These limitations should be discussed.

We share the reviewer's concern about pharmacological antagonists and now state this plainly in the discussion.

"FGFR pharmacological antagonists are also known to have off-target effects including on VEGF receptors and SRC."

The impairment of Closure 5 formation we observe is comparable between the pharmacological antagonists and genetic model we present.

5. The authors note that pERK1/2 persists on the PNP rim even after treating with BGJ398. While FGFRs are a major driver of ERK1/2 activity, there are numerous other RTKs that can drive ERK1/2 activity as well. The presence or absence of pERK1/2 therefore should not be used as sole evidence of FGFR activity or lack thereof. Based on efficacy in controls, perhaps an anti-pFGFR antibody could be used to verify the reduced activity of FGFRs in pharmacologically treated embryos.

© 2023. Published by The Company of Biologists under the terms of the Creative Commons Attribution License (https://creativecommons.org/licenses/by/4.0/).

We have now purchased and optimized a new p-FGFR1 antibody and used this to confirm a reduction of phosphorylation after 24 hours of treatment. This new data is now shown in Supplementary Figure 3 (below)

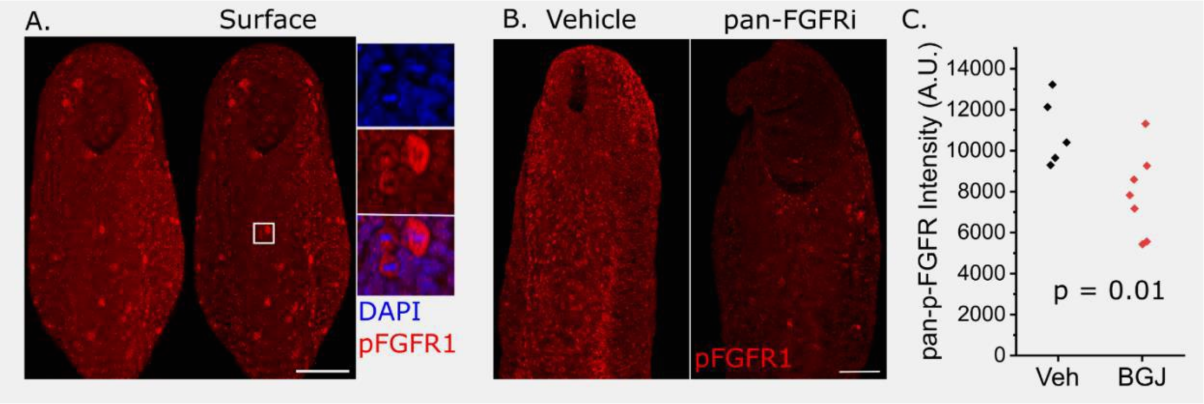

Supplementary Figure 3: Diminished p-FGFR1 following pharmacological antagonism. *A. Wholemount confocal image of a 28-somite embryo showing pFGFR1 immunolocalisation. The right panel is 'surface subtracted' to only show a 10 µm top surface, namely the surface ectoderm and apical neuroepithelium*. Insert shows bright pFGFR1 labelling mitotic cells.

B. 'Surface subtracted' wholemount confocal images of embryos cultured in vehicle or pan- FGFR inhibitor for 24 hours. Scale bar = 100 µm.

C. pFGFR1 immunofluorescence intensity quantification in the 'surface subtracted' surface ectoderm of control and pan-FGFR inhibitor (BGJ) treated embryos. Surface subtraction was used to ensure the volume analysed is comparable between Z-stacks. P value by t-test.

Minor Concerns
1.Similar neural tube closure defects have been reported in Fgfr1 mutants previously (PMID 317047;16421190; 4573858), although not thoroughly explored, reducing the novelty of these findings somewhat. The authors should cite these papers.

We have added these references but must emphasize that none of them carefully assessed neural tube development or Closure 5 formation (first descried in 2017).

2. The title of figure 2 "Tissue-specific functions of FGF signaling, likely through FGFR1, promote timely completion of PNP closure" instills a sense of vagueness. The statement should be clarified to be less ambiguous.

This title has been changed to read "Figure 2: FGF signalling is required for timely PNP closure."

3. The manuscript should be read through for typos, as a number are present:
a. Abstract line 2: classed -> classified
b. Into para 2 line 3: primary -> primarily
c. Results para 7 line 4-6: numbers are duplicated d. Results para 9 line 1: as -> a
e. Gene and protein names should be consistent for the species, as many mouse genes are not italicized properly, and clarification should be made as to whether a protein or gene is being discussed via proper nomenclature.

These corrections have been made.

<u>Reviewer 2 Comments for the Author:</u>
This is an interesting study which provides a careful description of closure 5 in mouse and human embryos. The main data of the paper involves experiments attenuating FGFR signalling using either

© 2023. Published by The Company of Biologists under the terms of the Creative Commons Attribution License (https://creativecommons.org/licenses/by/4.0/).

small molecule inhibitor or conditional genetic approach. These reveal complex phenotypes that while implicating FGFR signalling in this closure event also indicate multiple downstream events, for example, ectopic, mis-localised Sonic hedgehog signalling, which likely contribute to the generation of a myelocystocele-like morphology. There are also concerns about the precision of some experiments, which may contribute to complex phenotypes described here. Some specific points the authors may wish to consider for future revision include:

i) Experiments exposing embryos to the Pan FGFRi BGJ 398 (or PD 173074 mentioned in the Methods) for 24h appear not to have included assessment of the period when the inhibitor is active – data from cells in vitro suggests it is possible that this was for only 3-6h. Analysis of embryos in these experiments at 24h may then reflect phenotypes due to acute impact on morphological events, such as neurulation, while lack of apparent effect on other tissues such as somites is due to only transient loss of FGFR signalling? Along the same lines, it is difficult to interpret the dpERK detection at 24h in Fig2A, does this reflect action of resumed FGFR signalling, other RTK signalling pathways, or another ERK activating mechanism?

We thank the reviewer for this important point. It is not uncommon for the pharmacokinetics/dynamics of compounds used in whole embryo culture, with active circulation and distinct fluid compartments around the embryo, to differ from cell culture studies. We now provide three lines of evidence that the pan-FGFR inhibitor we used continues to be effective after 24 hours:

1. We provide new data showing phospho-FGFR1 levels are diminished in treated embryos (see Supplementary Figure 3 above). pFGFR1 does not follow the same staining pattern as pERK, supporting the suggestion that it is activated by different pathways.
2. The size of the penultimate somite, which would have formed a few hours before the end of culture, is significantly larger in FGFR-inhibited embryos as had previously been shown in the chick (see Supplementary Figure 4 above).
3. We have repeated analysis of the RARE domain in FGFR1-inhibited embryos much earlier, after 8 hrs (Supplementary Figure 6, below), to compare with the significant caudal expansion we had observed at 24 hours. RARE domain caudal expansion after 8 hrs was equivalent to that seen after 24 hours, again supporting the conclusion that the antagonist continues to inhibit FGFR at this late timepoint.

© 2023. Published by The Company of Biologists under the terms of the Creative Commons Attribution License (https://creativecommons.org/licenses/by/4.0/).

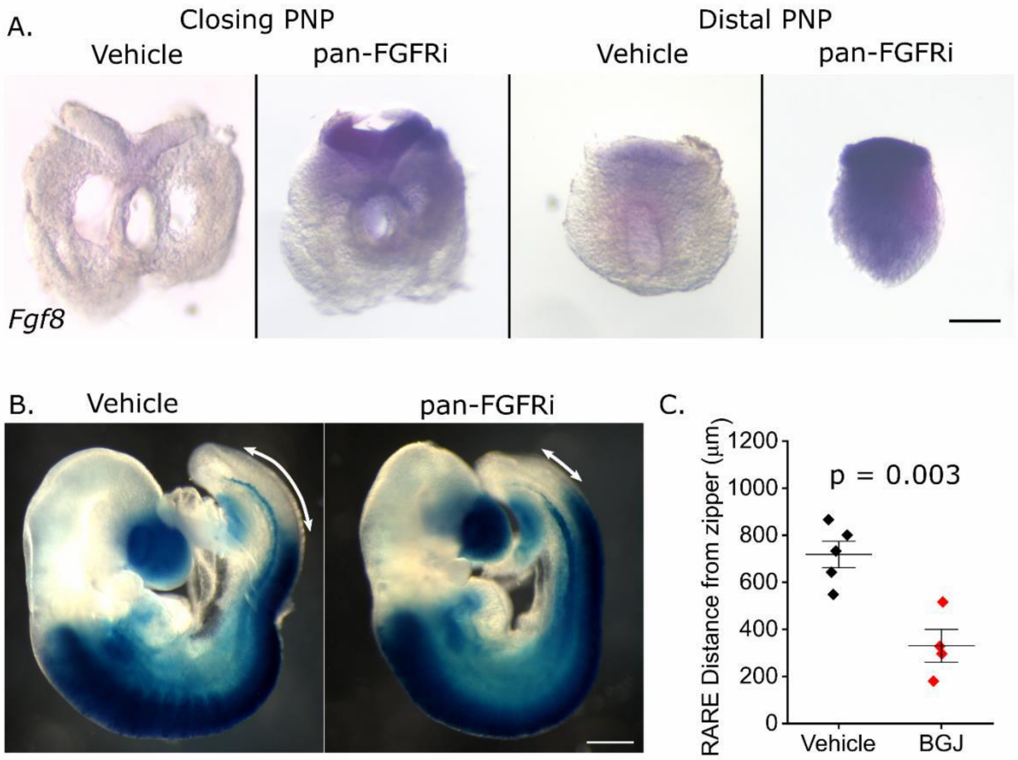

Supplementary Figure 6: Pan-FGFR inhibition for 8 hours increases Fgf8 and causes caudal expansion of RARE signalling.

A. Vibratome sections of wholemount Fgf8 in situ hybridisation in vehicle- and pan-FGFRi-treated embryos after 8 hours of whole embryo culture. Scale bar = 100 μm.

B. Brightfield images showing RARE-mediated LacZ expression domain in vehicle and pan- FGFRi-treated embryos after 24 hours of whole embryos culture. The double-headed white arrow indicates the distance between the RARE domain and the zippering point. Scale bar = 350 μm.
C. Quantification of RARE domain distance to the end of zippering point in vehicle and pan-FGFRi (BGJ)-treated embryos after 8 hours of whole embryo culture. Points represent individual embryos.

ii) Given that FGFR signalling promotes proliferation and in many contexts protects against cell death it would be informative to determine whether these fundamental processes, (which would affect tissue available for neurulation or for adjacent non-neural tissue providing biomechanical support) were affected following attenuation of this pathway.

We had only assessed proliferation and apoptosis in a preliminary way prior to submission and had not seen striking differences between genotypes. To fully address the reviewer's comment, we have now performed histological sectioning of embryos during the period of Closure 5 formation and confirmed findings in independent cohorts at two different developmental timepoints. These new analyses demonstrate *Fgfr1* deletion selectively diminishes proliferation of the neuroepithelium, potentially reducing its dorsal expansion to form Closure 5. This new data is now provided in Supplementary Figure 8.

© 2023. Published by The Company of Biologists under the terms of the Creative Commons Attribution License (https://creativecommons.org/licenses/by/4.0/).

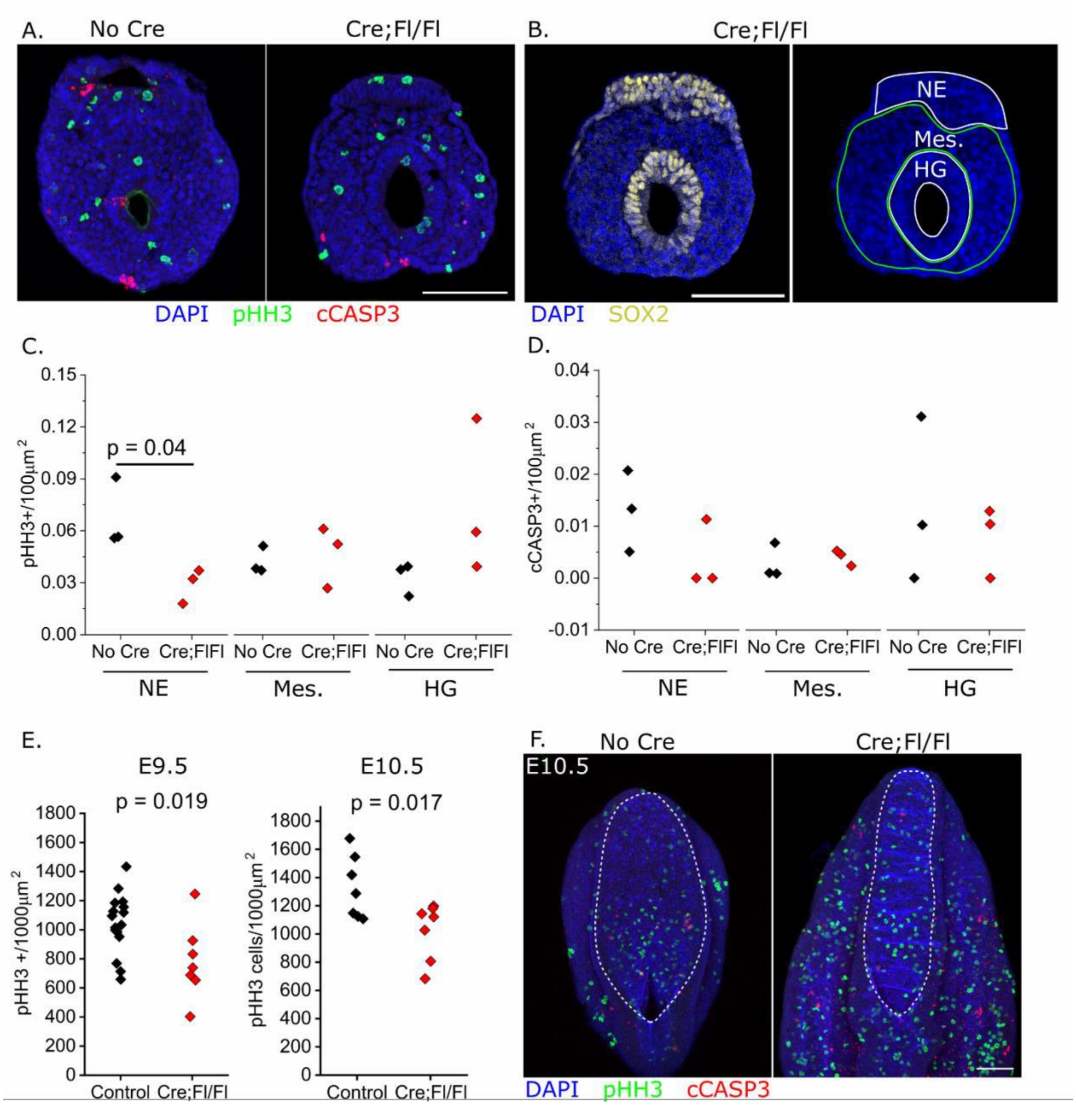

Supplementary Figure 8: Fgfr1 deletion diminishes neuroepithelial proliferation.
A-B*. Histological sections through the distal PNP of embryos at E10.5, when control embryos form Closure 5, immunofluorescently labelled to visualise the mitotic cell marker pHH3 and apoptosis marker cleaved caspase 3 (cCASP3).* ***B*** *shows annotation of the neuroepithelium (NE), mesoderm (Mes.) and hind-gut (HG) in an Fgfr1-deleted embryo immunolabelled for SOX2. Scale bars = 100 µm.*
C-D*. Quantification of the number of proliferating or apoptotic cells per area in the NE, mesoderm or HG. Points represent individual embryos (n = 3 embryos per genotype).*
E*. Quantification of the number of mitotic cells per area of exposed neuroepithelium in control and Fgfr1-deleted embryos collected at E9.5 or E10.5 (embryos with closed PNPs were excluded). Points represent individual embryos.*
F*. Wholemount immunofluorescent images showing proliferating and apoptotic cells in control and Fgfr1-deleted embryos at E10.5. The dashed white line indicates the exposed neuroepithelium. Scale bar = 100 µm.*

In fig2C the pan FGFRi embryo appears to lack caudal tissue. It is not clear from fig2B whether this is the case in this embryo assessed for Fgf8 expression. Sections of such embryos and some cell counts would be informative.

In situ was performed in embryos after 8 hours of culture, as specified in the figure legend, not 24 hours as shown in Fig 2c to visualise RARE. The highly corrugated NE after 24 hours of FGFR inhibition would risk in situ probe trapping. Sections through the embryos are now shown in Supplementary Figure 6, above.

© 2023. Published by The Company of Biologists under the terms of the Creative Commons Attribution License (https://creativecommons.org/licenses/by/4.0/).

iii) Figure 5D – ideally cell counts for neurons rather area in which TUJ1 expression is found is needed to conclude reduced neuron numbers. This can be done using a marker that localised to the cell body, for example HuC/D. For the TUJ1 data provided the numbers of sections and embryos sampled should be provided – I could not find this information in any of the figure legends nor in Methods, this should be provided for all quantifications in the paper – ideally in the figure legends.

We did not "conclude reduced neuron numbers", but rather that the "the TUJ1-stained area, relative to the area of visible neural tube, is greater in Cre;Fl/Fl embryos". We had been careful to describe this result accurately because hypoplasia of adjacent mesodermal tissues may make TUJ1-positive cells more readily visible. To fully address the reviewer's comment, we have performed HUC/D staining and counted the number of HUC/D positive cells relative to the number of SOX2-bright progenitors along a 100 µm length of the intermediate neural tube. This showed no difference between control and *Fgfr1*-deleted embryos. This supports our original conclusion that neuroepithelial "commitment to post-mitotic neurons is not diminished by loss of Fgfr1".

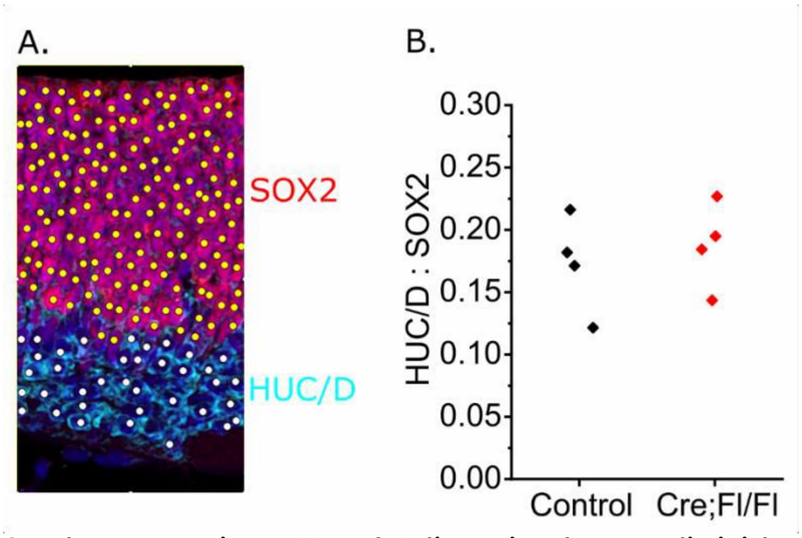

Supplementary Figure 11: Fgfr1 disruption does not diminish neurogenesis in the distal spine.
A. Representative image of a 100 µm length of the lateral neural tube stained and imaged equivalently to embryos in Figure 5G. Yellow dots indicate SOX2-bright cells, white dots indicate HUC/D-bright cells.
B. Quantification of the ratio of HUC/D to SOX2 bright cells along 100 µm lengths of the lateral neural tube in control and Fgfr1-disrupted embryos. Points represent individual embryos.

Throughout the manuscript, the embryo is the unit of measure and each point represents an individual embryo, expect when proportions are reported.

The discussion is wide ranging and raises further potential changes that may contribute to the complex phenotype that emerged from conditional Fgfr1 caudal loss. While these possibilities might be investigated to dissect distinct aspects of this FGFR signalling perturbation phenotype (e.g. ectopic Shh), this would require extensive further focussed investigation, which seems beyond the scope of this initial report and may not reveal a primary, initiating cause of this complex neurulation defect. The lack of clear mechanistic insight here leaves this a primarily descriptive study with no clear next steps for further insights. The authors have previously described closure 5 and this appears a largely incremental set of analyses, which while implicating FGFR1 also show that it is all much more complex.

Terminal Myelocystocele is a complex malformation which commonly presents with extra-spinal manifestations requiring urological, orthopaedic and neurological surgery. There is no reason to believe that its causation is simple, or could be recapitulated by disruption of one signalling pathway in a specific cell type. We have amended the discussion to clarify that previous reports of tissue-specific *Fgfr1* deletion did not describe equivalent neural tube

© 2023. Published by The Company of Biologists under the terms of the Creative Commons Attribution License (https://creativecommons.org/licenses/by/4.0/).

phenotypes. However, we agree with the reviewer's sentiment that "next steps" are often difficult without losing relevant multi-tissue interactions and resorting to reductionist approaches unlikely to capture clinically- relevant complexity. We hope to pursue both translational and mechanistic next steps building on our current findings, but in this substantial manuscript we now provide mechanistic insights including promotion of neuroepithelial proliferation by *Fgfr1* during Closure 5 formation and *Fgfr1*-dependant maintenance of dorsoventral progenitor domains selectively in the distal spine.

Reviewer 3 Comments for the Author:
I do not see that major revisions are needed. It is highly suitable for publication. My suggestion is to include more information regarding the source of human embryo material.

We thank the reviewer for their detailed reading of our manuscript and positive comments. Further details on HDBR material have now been added.

Second decision letter

MS ID#: DEVELOP/2023/202139

MS TITLE: Caudal Fgfr1 disruption produces localised spinal mis-patterning and a terminal myelocystocele-like phenotype in mice.

AUTHORS: Eirini Maniou, Faduma Farah, Abigail R Marshall, Zoe Crane-Smith, Andrea Krstevski, Athanasia Stathopoulou, Nicholas DE Greene, Andrew J. Copp, and Gabriel Galea

I have now received all the referees' reports on the above manuscript, and have reached a decision. The referees' comments are appended below, or you can access them online: please go to BenchPress and click on the 'Manuscripts with Decisions' queue in the Author Area.

As you will see, the referees express considerable interest in your work, but have some criticisms and recommend a further revision of your manuscript before we can consider publication. If you are able to revise the manuscript along the lines suggested, which may involve further experiments, I will be happy receive a revised version of the manuscript. Your revised paper will be re-reviewed by one or more of the original referees, and acceptance of your manuscript will depend on your addressing satisfactorily the reviewers' major concerns. Please also note that Development will normally permit only one round of major revision. If it would be helpful, you are welcome to contact us to discuss your revision in greater detail. Please send us a point-by-point response indicating your plans for addressing the referee's comments, and we will look over this and provide further guidance. In particular it may be that you consider some of the suggested experiments as being beyond what you consider "do-able" for the current paper - in which case please consider whether you might include these as future goals/experiments in the Discussion section of your paper.

Please attend to all of the reviewers' comments and ensure that you clearly highlight all changes made in the revised manuscript. Please avoid using 'Tracked changes' in Word files as these are lost in PDF conversion. I should be grateful if you would also provide a point-by-point response detailing how you have dealt with the points raised by the reviewers in the 'Response to Reviewers' box. If you do not agree with any of their criticisms or suggestions please explain clearly why this is so.

Reviewer 1

Advance summary and potential significance to field

© 2023. Published by The Company of Biologists under the terms of the Creative Commons Attribution License (https://creativecommons.org/licenses/by/4.0/).

The authors have addressed many of the concerns raised by this reviewer. They have now better assessed the period over which the FGFR inhibitor is active in their assays. They present new p-FGFR1 data that provides evidence for the effectiveness of the pan-FGFR inhibitor after 24h. It is curious that this is distinct from the dpERK detection pattern and the authors conclude that this suggests that it is activated by a distinct pathway, which may be the case. I would be a little cautious all the same, as dpERK activity is notoriously difficult to detect in a whole tissue context, and its induction by FGFR signalling is well established. Their findings could also reflect different phosphorylation stabilities and distinct regulatory feedback mechanisms rather than a different pathway.

A larger recently formed somite induced by the pan FGFR inhibitor is consistent with the literature from the Pourquie lab. but this morphology may reflect action at a much earlier time at the "determination front" and so does not necessarily provide evidence for inhibitor activity at 24h. It is curious that PD173074 did not generate the same somite size increase this could be due to different mechanism of action or inhibitor stability? the Pourquie lab used SU5402 which is less FGFR specific?

The data for expansion of the RARE-LacZ domain at 8h is consistent with an early effect of the inhibitor, I am not sure how this further supports its continued action at 24h.

The authors also provide further new data documenting the reduction of proliferation specifically in the neuroepithelium, while adjacent mesoderm is unaffected (and cell death is not increased in either tissue) following Fgfr1 deletion. This could potentially explain the reduced /delayed neural tube closure and provides further mechanistic insight. In this new data they refer to Fgfr1 deleted embryos – assuming these are cdx-cre driven Fgfr1 deletion embryos it might have been informative to have identified the cre expressing / Fgfr1 deleted cells in this assay although appreciate their comment that most cells express cdx-cre.

The authors have clarified their observations with respect to Sox2 and TUJ-1 expression and provide quantification of neurons using a further neuronal marker HuC/D. This new data now confirms that while neural progenitor proliferation is reduced, the number of neurons remains unchanged in following loss of Fgfr1.

Overall, these revisions increase assay precision and quantification of the data generated and provide more compelling evidence for a direct role for Fgfr1 in the regulation of the caudal neural tube closure.

Comments for the author

As above

<u>Reviewer 2</u>

Advance summary and potential significance to field

The manuscript by Galea et al., aims to describe the etiology of a myelocystocele-like phenotype arising from pharmacological or genetic inhibition of Fgfr1 in mouse. The authors focus on elucidating phenotypic changes in the Fgfr1 conditional knockout that precede the neural tube defect and therefore may be contributing to the etiology. Using imaging and subsequent quantitative analysis of pharmacological antagonism or regional genetic deletion of Fgfr1, the authors make several key findings (1) Humans have a similar sequence of posterior neuropore (PNP) closure with a possible closure 5 as inferred through brightfield images. (2) Fgfr1 is required for normal closure of the PNP. (3) Disruption of dorsal-ventral specification of the caudal neural tube precedes the myelocystocele-like phenotype in conditional Fgfr1 ko mouse embryos. These findings provide insight into the etiology of a specific caudal neural tube defect which are not often characterized in mouse. Following the consideration of the following revisions, this manuscript is suitable for Development and is of value to the community studying structural birth defects and specifically neural tube defects.

© 2023. Published by The Company of Biologists under the terms of the Creative Commons Attribution License (https://creativecommons.org/licenses/by/4.0/).

Comments for the author

The manuscript by Galea et al., aims to describe the etiology of a myelocystocele-like phenotype arising from pharmacological or genetic inhibition of *Fgfr1* in mouse. The authors focus on elucidating phenotypic changes in the *Fgfr1* conditional knockout that precede the neural tube defect and therefore may be contributing to the etiology. Using imaging and subsequent quantitative analysis of pharmacological antagonism or regional genetic deletion of *Fgfr1,* the authors make several key findings (1) Humans have a similar sequence of posterior neuropore (PNP) closure with a possible closure 5 as inferred through brightfield images. (2) *Fgfr1* is required for normal closure of the PNP. (3) Disruption of dorsal-ventral specification of the caudal neural tube precedes the myelocystocele-like phenotype in conditional *Fgfr1 ko* mouse embryos. These findings provide insight into the etiology of a specific caudal neural tube defect which are not often characterized in mouse. Following the consideration of the following revisions, this manuscript is suitable for *Development* and is of value to the community studying structural birth defects and specifically neural tube defects.

Major revisions:

While most experiments were carried out in the conditional genetic deletion, this reviewer thinks it is important to perform parallel characterization between pharmacological treatment and genetic deletion to be able to compare phenotypes of whole embryo vs regional restriction to better dissect contributions of *fgfr1*. In particular, this reviewer requests 3 key experiments:

1. Will reviewers test if there is similar retinoic acid expansion in the genetic deletion as in Figure
   2C. This can be with in situ hybridization for relevant genes driven by RAREs or retinoic acid receptors if crossing with the RARE-Lacz reporter line is too complicated or takes too much time.

    a. I ask about expansion of retinoic acid because in PMID 11157778 the authors shows in Fig 4C/F a similar thinning of dorsal cells and “trumpeting” of the lumen in *Cyp26A$^{-/-}$ KO* mouse embryos which should have an increased concentration of retinoic acid. In the referenced manuscript, the phenotype is of the “mild” variety, however it is like the conditional *fgfr1* deletion. Excess retinoic acid also leads to phenotypes like you see in your pharmacological perturbations, best shown in PMID 2190788, Fig 4C where embryos develop an outgrowth” or dramatic eversion of neural tissue in embryos treated with excess retinoic acid. Perhaps changes in retinoic acid domain expansion can explain one facet of discrepancies between phenotypes.

2. Do the embryos treated with FGFR1i 1) show dorsalization of the neural tube (or the outgrowth) and 2) do they present with “ventral ectopic clusters” expressing SHH and FOXA2? This experiment would require sectioning FGFR1i treated embryos and using immunofluorescence as in your current figure 6.

    a. The comparison of results from FGFR1i and *Fgfr1 KO* would be of interest to determine if global inhibition (which would be relevant to patients with an *Fgfr1* variant) also has a similar etiology to the specific function found by regional deletion.

3. Do the FGFR1i embryos have specific truncation of caudal elongation or is the forelimb to hindlimb growth also diminished? This experiment would be measuring FL-HL and HL-End in FGFR1i treated embryos as in Figure 3B to assess if loss global loss of *Fgfr1* has specific truncation or if the specific truncation has to do with the deletion being driven in a restricted region.

© 2023. Published by The Company of Biologists under the terms of the Creative Commons Attribution License (https://creativecommons.org/licenses/by/4.0/).

Second revision

Author response to reviewers' comments

We are pleased that the reviewers appreciate the value of our work and thank them for their comments which have helped us improve the manuscript further.

Reviewer 1:
The authors have addressed many of the concerns raised by this reviewer. They have now better assessed the period over which the FGFR inhibitor is active in their assays. They present new p-FGFR1 data that provides evidence for the effectiveness of the pan-FGFR inhibitor after 24h. It is curious that this is distinct from the dpERK detection pattern and the authors conclude that this suggests that it is activated by a distinct pathway, which may be the case. I would be a little cautious all the same, as dpERK activity is notoriously difficult to detect in a whole tissue context, and its induction by FGFR signalling is well established. Their findings could also reflect different phosphorylation stabilities and distinct regulatory feedback mechanisms rather than a different pathway.

We fully agree with the reviewer and have added this to the manuscript:

"Phosphorylation of ERK1/2 around the PNP rim persists despite FGFR inhibition (Figure 2A), suggesting activation through other mechanisms, different phosphorylation stabilities or distinct regulatory feedback mechanisms."

A larger recently formed somite induced by the pan FGFR inhibitor is consistent with the literature from the Pourquie lab. but this morphology may reflect action at a much earlier time at the "determination front" and so does not necessarily provide evidence for inhibitor activity at 24h. It is curious that PD173074 did not generate the same somite size increase, this could be due to different mechanism of action or inhibitor stability? the Pourquie lab used SU5402 which is less FGFR specific?

It is possible that this difference in phenotype may be due to pharmacokinetic differences between the antagonists used and state this in the manuscript, copied below. Our study also raises interesting questions about interactions between somites and the neural tube which we hope to pursue in future research.

"Absence of this somite phenotype when mouse embryos are cultured in the FGFR1- targeting antagonist used here may reflect differential tissue penetration, compound duration of action, or FGFR1-independent mechanisms in mice."

The data for expansion of the RARE-LacZ domain at 8h is consistent with an early effect of the inhibitor, I am not sure how this further supports its continued action at 24h.

The direct evidence we provide that FGFRs were antagonized after 24 hours of pharmacological treatment comes from the immunofluorescence analysis of pFGF1. Other lines of evidence simply support this. If the RARE domain had been more caudally expanded after 8 than 24 hours, we would have considered it likely that the compounds effects were waning at the later timepoint. That is not what we observe, providing an additional line of evidence that the FGFR inhibitor continued to have biological effects at the later timepoint.

The authors also provide further new data documenting the reduction of proliferation specifically in the neuroepithelium, while adjacent mesoderm is unaffected (and cell death is not increased in either tissue) following Fgfr1 deletion. This could potentially explain the reduced /delayed neural tube closure and provides further mechanistic insight. In this new data they refer to Fgfr1 deleted embryos – assuming these are cdx-cre driven Fgfr1 deletion embryos it might have been informative to have identified the cre expressing / Fgfr1 deleted cells in this assay although appreciate their comment that most cells express cdx-cre.

The reviewer raises an important point, which we address with new data, below, showing that

© 2023. Published by The Company of Biologists under the terms of the Creative Commons Attribution License (https://creativecommons.org/licenses/by/4.0/).

the vast majority of cells in the region analyzed have been recombined by Cdx2^{Cre}.

C. Cre;Fl/Fl

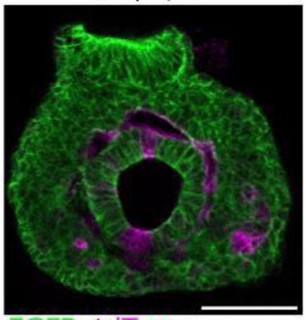

Supplementary Figure 8C: mTmG lineage tracing showing *Cdx2*^{Cre}-recombined (EGFP) and unrecombined (tdTom) cells in the distal tailbud of an E10.5 Cre;Fl/Fl embryo illustrating extensive lineage-tracing of most cells in this tissue. Scale bar = 100 µm.

The authors have clarified their observations with respect to Sox2 and TUJ-1 expression and provide quantification of neurons using a further neuronal marker HuC/D. This new data now confirms that while neural progenitor proliferation is reduced, the number of neurons remains unchanged in following loss of Fgfr1. Overall, these revisions increase assay precision and quantification of the data generated and provide more compelling evidence for a direct role for Fgfr1 in the regulation of the caudal neural tube closure.

No response needed.

Reviewer 2

Major revisions:

While most experiments were carried out in the conditional genetic deletion, this reviewer thinks it is important to perform parallel characterization between pharmacological treatment and genetic deletion to be able to compare phenotypes of whole embryo vs regional restriction to better dissect contributions of fgfr1. In particular, this reviewer requests 3 key experiments:

1. Will reviewers test if there is similar retinoic acid expansion in the genetic deletion as in Figure 2C. This can be with in situ hybridization for relevant genes driven by RAREs or retinoic acid receptors if crossing with the RARE-Lacz reporter line is too complicated or takes too much time.

a. I ask about expansion of retinoic acid because in PMID 11157778 the authors shows in Fig 4C/F a similar thinning of dorsal cells and "trumpeting" of the lumen in Cyp26A-/- KO mouse embryos which should have an increased concentration of retinoic acid. In the referenced manuscript, the phenotype is of the "mild" variety, however it is like the conditional fgfr1 deletion. Excess retinoic acid also leads to phenotypes like you see in your pharmacological perturbations, best shown in PMID 2190788, Fig 4C where embryos develop an outgrowth" or dramatic eversion of neural tissue in embryos treated with excess retinoic acid. Perhaps changes in retinoic acid domain expansion can explain one facet of discrepancies between phenotypes.

We agree with the reviewer that embryos we cultured in FGFR inhibitors show exciting similarities with previous studies of excess retinoic acid signaling (at early stages these are probably best shown in PMID 2014479). Our analyses of the RARE domain led us to conclude that this did not extend into the open neuropore and is therefore unlikely to cause the neuroepithelial abnormalities and lack of Closure 5 observed. We had also presented in situ data of a retinoic acid response gene, *Fgf8*, whose caudal expression is inhibited by retinoic acid (Kumar and Duester Dev. 2014). In our pharmacological inhibition models, *Fgf8* expression is up-regulated in the neuropore, further supporting our conclusion that retinoic signaling is not over-active in this tissue.

© 2023. Published by The Company of Biologists under the terms of the Creative Commons Attribution License (https://creativecommons.org/licenses/by/4.0/).

We have now performed additional in situ experiments in our conditional transgenic embryos, also observing a larger caudal *Fgf8* domain (shown below). We intend to continue working towards an understanding of how abnormal interactions between FGF and RA signalling contribute to spinal dysraphism beyond this study. Our hypothesis is that some phenotypes caused by excess RA involve inhibition of FGF signalling, resulting in similarities with our FGF- inhibition models.

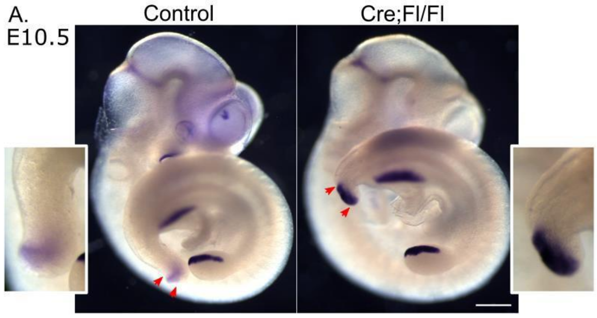

Supplementary Figure 9A: Wholemount *Fgf8* in situ hybridisation in control and littermate Cre;Fl/Fl embryos. Arrowheads indicate the tailbud *Fgf8* domain, shown in the insets. Scale bar = 300 µm.

With regards to the neural patterning phenotypes, we had suggested the following: “One possible explanation for this regional phenotype is interactions with adjacent mesodermal structures, which are particularly abnormal caudally in our model. This is consistent with lack of sclerotome-derived vertebral bodies below the cystic lesion of *Fgfr1*-disrupted fetuses. Sclerotome has been suggested to act both as a conduit for SHH diffusion as well as being a target tissue for its action (59) and somite-derived retinoic acid is mutually antagonistic with FGF signalling in regulating caudal neurogenesis (16).”

In support of this, we have now done additional experiments to localise the retinoic acid synthesizing enzyme RALDH2 (shown below). In control embryos, RALDH2 immunolocalises strongly in somites and less abundantly in other tissues. Conditional *Fgfr1* deleted embryos show less RALDH2 within hypoplastic somites at the level of the hindlimbs, but more intense, diffuse localization in the dorsal neural tube and adjacent mesenchyme. Thus, the later phenotypes we observe are unlikely to simply result from increased RA signalling, but may involve altered distribution and timing of RA exposure, as is now discussed:

“The combination of diminishing retinoic acid production by somites adjacent to the closed neural tube, and loss of repression by FGF signaling, suggests complex changes in the source, timing and intensity of neuroepithelial retinoic acid exposure in our model.”

© 2023. Published by The Company of Biologists under the terms of the Creative Commons Attribution License (https://creativecommons.org/licenses/by/4.0/).

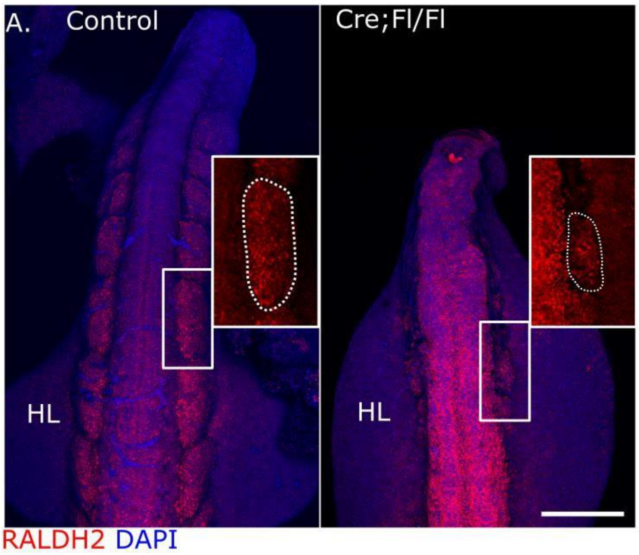

Supplementary Figure 10A: Wholemount immunofluorescent visualization of RALDH2 in an E10.5 control and littermate Cre;Fl/Fl embryo. Inserts show magnified views of a somite in each embryo (dashed lines). HL = hindlimbs, scale bar = 250 µm.

2. Do the embryos treated with FGFR1i 1) show dorsalization of the neural tube (or the outgrowth) and 2) do they present with "ventral ectopic clusters" expressing SHH and FOXA2? This experiment would require sectioning FGFR1i treated embryos and using immunofluorescence as in your current figure 6. a. The comparison of results from FGFR1i and Fgfr1 KO would be of interest to determine if global inhibition (which would be relevant to patients with an Fgfr1 variant) also has a similar etiology to the specific function found by regional deletion.

Our culture model is only extended to early E10.5 (<30 somites), before most of the patterning changes we describe in the conditional knockout are observed. We do see ectopic clusters and ventral extension of the OLIG2 domain in a subset of conditional knockout embryos at E10.5, and have therefore performed additional experiments to assess these phenotypes in four FGFR-inhibited embryos.

We did not observe ectopic clusters or ventral extension of OLIG2 (shown below for the reviewer's information) in these embryos. We cannot exclude these phenotypes emerging at more caudal positions, which are still open in the FGFR-inhibited embryos at the stages cultured to, or they may only emerge in a subset of embryos as seen in the genetic model. This analysis does not meaningfully inform our conclusions, and we therefore do not feel it should be included. It is also possible that they require persistent FGFR1 signalling in the notochord (which is not recombined in our genetic model), but this requires future testing as is now discussed:

"We observe that *Cdx2*^{Cre} does not recombine in the notochord: whether persistent FGFR1 signalling in this tissue contributes to the phenotypes observed remains to be determined."

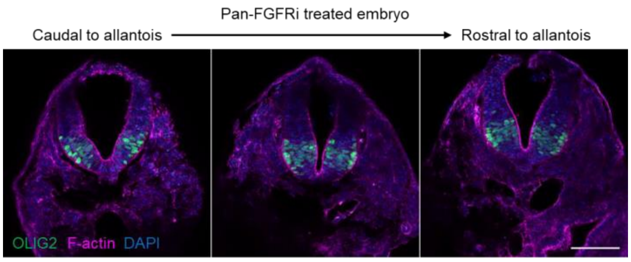

Figure provided for the reviewer's information: Serial sectioning of an embryo cultured in pan-FGFR inhibitor immunolabelled to visualize the OLIG2 domain around the level of the allantois. Note the OLIG2-negative floorplate in all three sections. Scale bar = 100 µm.

© 2023. Published by The Company of Biologists under the terms of the Creative Commons Attribution License (https://creativecommons.org/licenses/by/4.0/).

3. Do the FGFR1i embryos have specific truncation of caudal elongation or is the forelimb to hindlimb growth also diminished? This experiment would be measuring FL-HL and HL-End in FGFR1i treated embryos as in Figure 3B to assess if loss global loss of Fgfr1 has specific truncation or if the specific truncation has to do with the deletion being driven in a restricted region.

We have measured these lengths as requested by the reviewer and provide them for their information below. Similarly to our observations in the conditional knockout embryos, we observe reduced body axis length caudal to the hindlimb buds but not between the forelimb and hindlimb buds in embryos cultured for 24 hours in FGFR pharmacological inhibitor. However, in these experiments the inhibitor is added acutely after most of the anterior body axis had already formed (unlike the early Cre driver used). We therefore do not wish to draw direct conclusions from this data and have not included them in the manuscript.

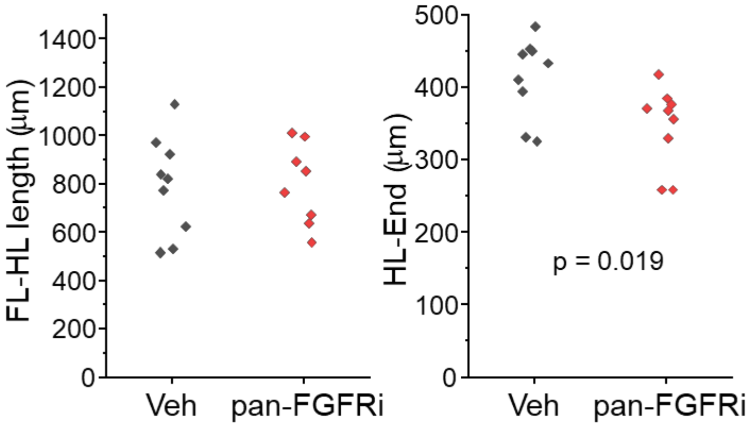

Figure provided for the reviewer's information: Quantification of the lengths between the forelimb and hindlimb (at the level of the allantois) or hindlimb to tail end in embryos cultured in vehicle of pan-FGFR inhibitor for 24 hours. Points represent individual embryos, P value by unpaired T-test.

Third decision letter

MS ID#: DEVELOP/2023/202139

MS TITLE: Caudal Fgfr1 disruption produces localised spinal mis-patterning and a terminal myelocystocele-like phenotype in mice.

AUTHORS: Eirini Maniou, Faduma Farah, Abigail R Marshall, Zoe Crane-Smith, Andrea Krstevski, Athanasia Stathopoulou, Nicholas DE Greene, Andrew J. Copp, and Gabriel Galea

I have now received all the referees reports on the above manuscript, and have reached a decision. The referees' comments are appended below, or you can access them online: please go to BenchPress and click on the 'Manuscripts with Decisions' queue in the Author Area.

The overall evaluation is positive and we would like to publish your manuscript in Development. As you will see, Reviewer 1 has suggested the inclusion of a further reference that they feel is relevant to your work. Hopefully it will only take you a couple of minutes to amend the word file before uploading the very final version. Your paper will not require any further review rather I shall accept it once it reappears on the website.

© 2023. Published by The Company of Biologists under the terms of the Creative Commons Attribution License (https://creativecommons.org/licenses/by/4.0/).

Reviewer 1

Advance summary and potential significance to field

Advances as summarised previously.

Comments for the author

The authors have satisfactorily addressed comments raised by this reviewer.
In reading the paper through again the authors appear to have over-looked a relevant paper in the chick embryo, which address FGF/RA interactions and ventral patterning neural, FGF/Shh regulation, which may help to place their findings in context - https://pubmed.ncbi.nlm.nih.gov/14527434/. It would be appropriate to address this in the discussion.

Reviewer 2

Advance summary and potential significance to field

The manuscript by Galea et al., aims to describe the etiology of a myelocystocele-like phenotype arising from pharmacological or genetic inhibition of Fgfr1 in mouse. The authors focus on elucidating phenotypic changes in the Fgfr1 conditional knockout that precede the neural tube defect and therefore may be contributing to the etiology. Using imaging and subsequent quantitative analysis of pharmacological antagonism or regional genetic deletion of Fgfr1, the authors make several key findings (1) Humans have a similar sequence of posterior neuropore (PNP) closure with a possible closure 5 as inferred through brightfield images. (2) Fgfr1 is required for normal closure of the PNP. (3) Disruption of dorsal-ventral specification of the caudal neural tube precedes the myelocystocele-like phenotype in conditional Fgfr1 ko mouse embryos. These findings provide insight into the etiology of a specific caudal neural tube defect which are not often characterized in mouse. This manuscript is suitable for Development and is of value to the community studying structural birth defects and specifically neural tube defects.

Comments for the author
This reviewer thanks the authors for their responses and has no further revision requests.

Third revision

Author response to reviewers' comments

The additional reference requested by the reviewer has now been added to the manuscript.

Fourth decision letter

MS ID#: DEVELOP/2023/202139

MS TITLE: Caudal Fgfr1 disruption produces localised spinal mis-patterning and a terminal myelocystocele-like phenotype in mice.

AUTHORS: Eirini Maniou, Faduma Farah, Abigail R Marshall, Zoe Crane-Smith, Andrea Krstevski, Athanasia Stathopoulou, Nicholas DE Greene, Andrew J. Copp, and Gabriel Galea
ARTICLE TYPE: Research Article

© 2023. Published by The Company of Biologists under the terms of the Creative Commons Attribution License (https://creativecommons.org/licenses/by/4.0/).

I am happy to tell you that your manuscript has been accepted for publication in Development, pending our standard ethics checks.

© 2023. Published by The Company of Biologists under the terms of the Creative Commons Attribution License (https://creativecommons.org/licenses/by/4.0/).
